# Schwann Cell Stimulation of Pancreatic Cancer Cells: A Proteomic Analysis

**DOI:** 10.3389/fonc.2020.01601

**Published:** 2020-08-25

**Authors:** Aysha Ferdoushi, Xiang Li, Nathan Griffin, Sam Faulkner, M. Fairuz B. Jamaluddin, Fangfang Gao, Chen Chen Jiang, Dirk F. van Helden, Pradeep S. Tanwar, Phillip Jobling, Hubert Hondermarck

**Affiliations:** ^1^School of Biomedical Sciences and Pharmacy, Faculty of Health and Medicine, University of Newcastle, Callaghan, NSW, Australia; ^2^Hunter Medical Research Institute, University of Newcastle, New Lambton, NSW, Australia; ^3^Department of Biotechnology and Genetic Engineering, Mawlana Bhashani Science and Technology University, Tangail, Bangladesh; ^4^School of Medicine and Public Health, The University of Newcastle, Callaghan, NSW, Australia

**Keywords:** pancreatic cancer, Schwann cells, secretome, cancer cell proliferation and invasion, LC-MS/MS, therapeutic targets

## Abstract

Schwann cells (SCs), the glial component of peripheral nerves, have been identified as promoters of pancreatic cancer (PC) progression, but the molecular mechanisms are unclear. In the present study, we aimed to identify proteins released by SCs that could stimulate PC growth and invasion. Proteomic analysis of human primary SC secretome was performed using liquid chromatography–tandem mass spectrometry, and a total of 13,796 unique peptides corresponding to 1,470 individual proteins were identified. Gene Ontology and Kyoto Encyclopedia of Genes and Genomes pathway enrichment were conducted using the Database for Annotation, Visualization, and Integrated Discovery. Metabolic and cell–cell adhesion pathways showed the highest levels of enrichment, a finding in line with the supportive role of SCs in peripheral nerves. We identified seven SC-secreted proteins that were validated by western blot. The involvement of these SC-secreted proteins was further demonstrated by using blocking antibodies. PC cell proliferation and invasion induced by SC-conditioned media were decreased using blocking antibodies against the matrix metalloproteinase-2, cathepsin D, plasminogen activator inhibitor-1, and galectin-1. Blocking antibodies against the proteoglycan biglycan, galectin-3 binding protein, and tissue inhibitor of metalloproteinases-2 decreased only the proliferation but not the invasion of PC cells. Together, this study delineates the secretome of human SCs and identifies proteins that can stimulate PC cell growth and invasion and therefore constitute potential therapeutic targets.

## Introduction

Pancreatic cancer (PC) is one of the most lethal malignancies (1) and is predicted to become the second leading cause of cancer-related death by 2030 (2). Eighty-five percent of PC cases are ductal adenocarcinomas with a 5-year survival rate less than 7% (3). The poor patient survival is attributed to late-stage diagnosis, high incidence of local recurrence, development of distant metastases, and therapeutic resistance (4, 5). In addition, there is currently no targeted therapy for PC, and therefore the identification of potential therapeutic targets is essential.

Schwann cells (SCs) are the major glial component in the peripheral nervous system (6). SCs maintain neuronal homeostasis through the regulation of cell growth, survival, and repair (7, 8). The primordial role of SCs is myelination (9), however SCs have recently been implicated in several malignancies including pancreatic (10–12), prostate (13), lung (14), oral (15), and cervical (16) cancers. In PC, SCs are involved in the initiation of disease, and their presence is associated with increased perineural invasion, the process by which cancer cells invade nerves (10). SCs guide cancer cells toward nerves via the production of neural cell adhesion molecule 1 (NCAM 1) that promotes perineural invasion (11). SCs have also been shown to initiate epithelial–mesenchymal transition and support metastatic spread (12), and SCs have been reported to mask cancer-related pain, resulting in a prolonged asymptomatic phase and delayed cancer diagnosis (17). Additionally, SC-derived interleukin 6 has been reported to augment PC cell migration and invasion (18). Although few SC-secreted cytokines (19) and adhesion molecules (20) have been described, the secretome of SC and its impact in PC remain largely unknown.

In the present study, we have profiled the secretome of human SCs using liquid chromatography–tandem mass spectrometry (LC-MS/MS) and investigated the role of several identified proteins in the stimulation of PC growth and invasion. These secreted proteins may constitute new therapeutic targets for PC.

## Materials and Methods

### Human SC Culture

Primary human SCs, obtained from the spinal nerve cells of a healthy donor, were purchased from ScienCell (cat. no. 1700, CA, United States) and maintained (maximum of 10 passages) according to manufacturer instructions described previously (21). Briefly, T-75 culture flasks were coated with 10 mg/mL poly-L-lysine (cat. no., 0413, ScienCell) and incubated overnight at 37°C. Cells were seeded at 5,000 cells/cm^2^ on the poly-L-lysine–coated flask after washing the vessel twice with sterile milli-Q water. Cells were grown in complete SC medium (SCM, cat. no., 1701, ScienCell), supplemented with 5% fetal bovine serum (FBS, cat. no., 0025, ScienCell), 1% SC growth supplement cocktail (SCGS, cat. no., 1752, ScienCell), and 1% penicillin/streptomycin (P/S, cat. no., 0503, ScienCell) in a humidified incubator at 37°C with 5% CO_2_. SCs were characterized by immunoblotting using antibodies against human SC marker proteins, SOX10, and p75 (Supplementary Figure S1).

Pancreatic ductal adenocarcinoma cells, PANC-1 and MIA PaCa-2, were obtained from the American Type Culture Collection (ATCC, Manassas, VA, United States) and maintained in Dulbecco modified eagle medium (cat. no., ATC302002, ATCC) supplemented with 10% (vol/vol) FBS (JRH Biosciences, St. Louis, MO, United States) and 2 mM L-glutamine in a humidified incubator at 37°C with 5% (vol/vol) CO_2_.

### SC Conditioned Media Preparation

**FIGURE 1:**
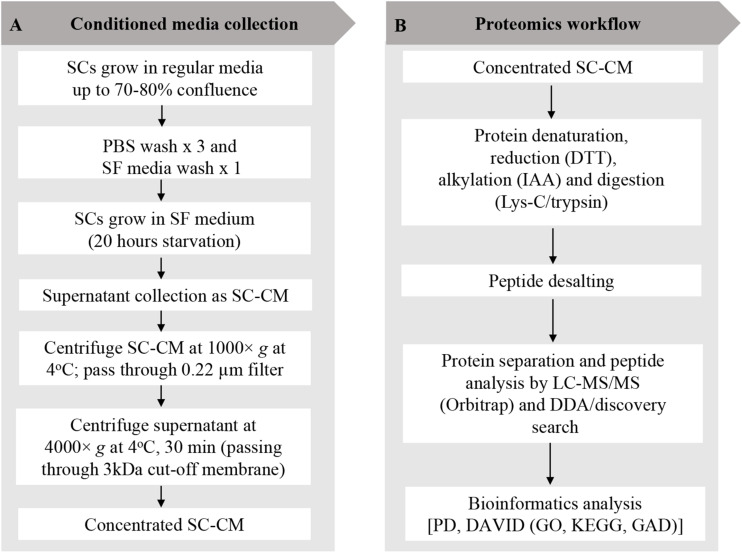
Schwann cell–conditioned media (SC-CM) collection and proteomic workflow. **(A)** For SC-CM collection, SCs were grown to 70–80% confluence. Cells were washed three times with sterile PBS and once with SF media before incubation in SF media for additional 20 h. SC-CM were then collected and centrifuged (1,000 × *g*, 4°C, 10 min), and the supernatant was filtered through a 0.22-μm nylon filter to remove any cell debris or floating cells. Collected supernatant was concentrated using 3-kDa cutoff Amicon Ultra-15 filter unit (4,000 × *g*, 4°C, 30 min). **(B)** Proteomic analysis of the secretome was performed using LC-MS/MS. Concentrated SC-CM was subjected to reduction (DTT), alkylation (IAA), and Lys-C/trypsin digestion before analysis in LC-MS/MS. DDA discovery search was performed to identify the total number of peptides and proteins. To profile the SC-secretome, functional clustering of the secreted proteins was performed using DAVID bioinformatics software and displayed in GO and KEGG. Identified proteins were classified based on disease classes using DAVID-GAD analysis. SC, Schwann cell; PBS, phosphate-buffered saline; SF, serum-free; SC-CM, Schwann cell–conditioned media; DTT, dithiothreitol; IAA, iodoacetamide; LC-MS/MS, liquid chromatography–tandem mass spectrometry; DDA, data-dependent acquisition; PD, Proteome Discoverer; DAVID, Database for Annotation, Visualization, and Integrated Discovery; GO, Gene Ontology; KEGG, Kyoto Encyclopedia of Genes and Genomes; GAD, Genetic Association Database.

SCs were grown to 70–80% confluency in SCM and washed three times with sterile phosphate-buffered saline (PBS) (Invitrogen, CA, United States) and once with serum-free (SF) media. SCs were then incubated in SF media for 20 h, after which SC-conditioned media (SC-CM) was collected, centrifuged (1,000 × *g* at 4°C for 10 min), and the supernatant was filtered through a 0.22-μm nylon filter (Merck Millipore, MA, United States) to remove any cell debris or floating cells. SC-CM was further centrifuged (4,000 × *g* at 4°C for 30 min) to concentrate using a 3-kDa cutoff Amicon Ultra-15 filter unit (Merck Millipore) until the media was concentrated 400-fold. The recovered SC-CM concentrate was stored at −80°C. An outline of SC-CM collection and concentration workflow are shown in Figure 1A.

### Mass Spectrometry–Based Proteomic Analysis of SC Secretome

Discovery proteomic analysis was performed by LC-MS/MS to describe the secretome profile of primary human SCs. Experimental protocol outlined in Figure 1B has been previously described (21) with additional sample preparation steps added for the secretome analysis. The secretome of SCs have been analyzed in an unstimulated state, i.e., without previous exposure to PC cells.

### Sample Preparation for LC-MS/MS

Two hundred micrograms of secreted proteins was measured by bicinchoninic acid (BCA) assay (Pierce, Thermo Fisher Scientific, IL, United States) and dissolved in urea (6 M urea 2 M thiourea) buffer followed by reduction step using 10 mM dithiothreitol (30 min at room temperature). The samples were subsequently alkylated using 20 mM iodoacetamide (30 min at room temperature in the dark). Proteins were digested using 1:40 ratio Lys-C/trypsin (cat. no., VA1170, Promega, Madison, WI, United States) to protein concentration (3 h, room temperature, in the dark). The concentration of the urea was brought down to less than 1 M by adding 20 mM triethylammonium bicarbonate (pH 7.8) and incubated overnight at room temperature. Peptides were desalted and cleaned up using a Visiprep^TM^ vacuum manifold (12-port, cat. no. 57030-U, Sigma-Aldrich, St. Louis, MO, United States) coupled with Empore C18 solid-phase extraction cartridge (4 mm/1 mL) according to manufacturer instructions.

### LC-MS/MS

A Dionex UltiMate 3000 nanoLC system (Thermo Fisher Scientific) with a 15-cm EASY-Spray Column was used to separate 500 ng of peptides using a 150-min gradient at a flow rate of 300 nL/min. Peptides were analyzed on Q-Exactive Plus Orbitrap (Thermo Fisher Scientific) mass spectrometer. Precursor scan of intact peptides was measured in the Orbitrap by scanning from m/z 400–2,000, with a resolution of 70 K with maximum ion injection time of 50 ms and automated gain control (AGC) target of 1E6. The 15 most intense multiply charged precursors were selected for HCD fragmentation with a normalized collision energy of 27.0 and then measured in the Orbitrap at a resolution of 35 K with maximum ion injection time of 120 ms, and AGC target was set at 2E5. Dynamic exclusion was set for 30 s.

### MS Data Analysis

LC-MS/MS data were analyzed using Proteome Discoverer software v.2.1 (Thermo Fisher Scientific) and searched against the Uniprot human protein database (downloaded March 5, 2018, with a total of 71,773 entries). Precursor mass tolerance was set to 10 ppm, and fragment ion tolerance was 0.02 Da. Trypsin was designated as the digestion enzyme with two missed cleavages permitted. Carbamidomethylation on cysteine (+57.021 Da) was set as static modifications, and oxidation on methionine (+15.995 Da) was set as variable modification. Only high confidence identification, represented by at least two unique peptides, was included in the analysis. Protein confidence indicators were set at 1% false discovery rate criteria using a percolator. The precursor ion (MS) spectra were also manually validated using Xcalibur Software version 4.0.27.13 (Thermo Fisher Scientific) to avoid false-positive detection.

### Proliferation Assay

Pancreatic cancer cells (PANC-1 and MIA PaCa-2) were starved for 24 h and seeded (∼5,000 cells/well) in a 96-well culture plate. Cells were cultured in 200 μL SF media, serum-supplemented media (noted as FBS), and SC-CM, with or without specific blocking antibodies (6 μg/mL) (detailed information of the antibodies is listed in Table 1). Antibodies were used to block specific proteins in SC-CM. Of note, the commercial producers have reported blocking activity for the antibodies that we have used, but we have not independently confirmed it. Cells were cultured for 72 h and subsequently incubated with CellTiter-Blue^®^ (Promega Corporation, cat. no., G8081) at 37°C for 4 h before recording fluorescence at 560/590 using a FLUOstar OPTIMA fluorescence plate reader (BMG Labtech, Durham, NC, Untied States). All the experiments were repeated three times.

**TABLE 1:** Antibodies used for WB.

No.	Antibody name	Source (working dilution)	Company (cat. no.)
**Antibodies used to detect protein in SC-CM**			
1	Gal-3BP	Goat polyclonal (1:700)	R&D System (AF2226)
2	MMP-2	Goat polyclonal (1:500)	R&D System (AF902)
3	Cathepsin D	Goat polyclonal (1:100)	R&D System (AF1014)
4	PAI-1	Goat polyclonal (1:100)	R&D System (AF1786)
5	Biglycan	Goat polyclonal (1:1,000)	R&D System (AF2667)
6	TIMP-2	Goat polyclonal (1:500)	R&D System (AF971)
7	Gal-1	Goat polyclonal (1:1,000)	R&D System (AF1152)
**Antibodies used to detect protein in cell lysate**			
8	Sox10	Mouse monoclonal (1:400)	Abcam (ab212843)
9	p75	Rabbit polyclonal (1:200)	Santa Cruz, (H-137): sc-8317
**Antibodies used to detect loading control**			
10	β-actin	Mouse monoclonal (1:2,000)	Abcam (ab8226)
11	β-actin	Rabbit polyclonal (1:2,000)	Abcam (ab8227)

*Antibodies used to detect proteins both in SC-CM and cell lysate are presented here with source, dilution, company name, and cat. no. SC-CM, Schwann cell–conditioned media; Gal-3BP, galectin-3 binding protein; MMP-2, matrix metalloproteinase-2; PAI-1, plasminogen activator inhibitor-1; TIMP-2, tissue inhibitor of metalloproteinases-2; Gal-1, galectin-1; cat. no., catalog number.*

### Invasion Assay

Cell invasion assays were performed on serum-starved PC cells (PANC-1 and MIA PaCa-2) using the QCM ECM Cell Invasion Assay kit (cat. no., ECM554; Merck Millipore). The supplied 24-well assay plate contains upper invasion chamber inserts with 8-mm pore size membranes. The extracellular matrix (ECM) layer was rehydrated with 300 μL of prewarmed SF media for 30 min at room temperature. Serum-starved cells (∼60,000) were loaded into the Transwell chamber insert in 250 μL of SF media or SC-CM with or without specific antibody (6 μg/mL). Five hundred microliters of SF medium, serum-supplemented media (noted as FBS), and SC-CM, with or without specific antibody (6 μg/mL), was added to the lower chamber. After 24 h, invading cells were dislodged, and the fluorescence was recorded at 480/520 nm using a FLUOstar OPTIMA fluorescence plate reader (BMG Lab-tech) as described previously (22). All the experiments were repeated three times.

### Western Blotting

Concentrated SC-CM and cellular lysates underwent western blot (WB) analysis to detect specific proteins of interest from LC-MS/MS in the SC secretome and SCs, respectively. After collecting the SC-CM for secretome profiling and functional analysis, the remaining cells were washed three times with PBS, trypsinized, and collected by gently scrapping into PBS media. Cells were subsequently pelleted by centrifugation at 800 × *g* for 5 min. Total proteins were extracted from cell pellets using RIPA buffer [25 mM Tris–HCl (pH 7.6)], 150 mM NaCl, 1% NP-40, 1% sodium deoxycholate, 0.1% sodium dodecyl sulfate (SDS)] (Thermo Fisher Scientific) and commercial protease inhibitor and phosphatase inhibitor cocktail tablets (Roche, Mannheim, Germany), aliquoted, and stored at −20°C. The total protein concentration of cell extracts and concentrated SC-CM was determined using a BCA assay (Pierce), according to the manufacturer’s instructions.

Thirty micrograms of protein from each sample was resuspended in an equal volume of Laemmli buffer (Bio-Rad, Hercules, CA, United States). The cell extract or concentrated SC-CM was subjected to SDS–polyacrylamide gel electrophoresis under reducing conditions, and the separated proteins were transferred to 0.4-mm pore nitrocellulose membranes (Amersham, GE Healthcare Life Sciences, Pittsburgh, PA, United States). Blots were blocked with blocking buffer (LI-COR Biosciences, Lincoln, NE, United States) for 1 h at room temperature and then probed with antibodies against specific proteins (Table 1). Identical antibodies were used for both WB and functional analysis. β-Actin protein expression was used as loading control. All antibodies were diluted in blocking buffer (LI-COR Biosciences). After washing with PBS containing 0.1% Tween-20, membranes were probed with goat anti–mouse or goat anti–rabbit IR-Dye 670 or 800 cw labeled secondary antisera, and then washes were repeated after labeling. WB was imaged using the LI-COR Odyssey infrared imaging system (LI-COR Biosciences).

### Pancreatic Tissue Samples and Immunohistochemistry

High-density tumor micro arrays (TMAs) were obtained from US Biomax Inc. (Maryland, MD, United States). The TMAs used (HPan-Ade170Sur-01) included a total of 99 pancreatic adenocarcinomas and 71 normal adjacent pancreatic tissues. For each specimen collected, informed consent was obtained from both the hospital and the individual. Discrete legal consent was obtained, and the rights to hold research uses for any purpose or further commercialized uses were waived. The study was approved by the University of Newcastle’s Human Research Ethics Committee.

Immunohistochemistry (IHC) was performed as described previously (23). Following deparaffinization and rehydration of the TMA slides using standard procedures, heat-induced epitope retrieval was carried out in a low-pH, citrate-based antigen unmasking solution (catalog number H-3300, Vector Laboratories, California, CA, United States) by a decloaking chamber (Biocare, West Midlands, United Kingdom) at 95°C for 30 min and 90°C for 10 s. IHC was then performed using an ImmPRESS^TM^ horseradish peroxidase (HRP) immunoglobulin G (peroxidase) Polymer Detection Kit (Vector Laboratories), as per the manufacturer’s recommendations. After inactivation of endogenous peroxidases with 0.3% H_2_O_2_ and blocking with 2.5% horse serum, primary antibody followed by secondary antibodies was applied to the sections and revealed with DAB peroxidase (HRP) Substrate Kit (catalog number SK-4100, Vector Laboratories). Primary antibodies used are listed in Supplementary Table S1. Finally, TMA slides were counterstained with hematoxylin (Gill’s formulation, Vector Laboratories), dehydrated, and cleared in xylene before mounting in Ultramount #4 mounting media (Thermo Fisher Scientific, Victoria, Australia). Following IHC staining, slides were scanned with a Leica Aperio AT2 Scanner (Leica Biosystems, Vista, CA, United States) (23).

### Bioinformatics Analysis

UniProt was used to identify protein cellular localization. Functional clustering of the identified secreted proteins was performed using the Database for Annotation, Visualization, and Integrated Discovery (DAVID, v6.8^1^) searching against the entire Homo sapiens genome (access date, March 25, 2020). DAVID classified the characteristic protein sets according to Gene Ontology (GO) terms for cellular compartments, biological processes, and molecular functions. DAVID was also used to recognize functional Kyoto Encyclopedia of Genes and Genomes (KEGG) pathway categories. All the DAVID categories were ranked according to the number of proteins in each group and not with *p*-value. To investigate the potential associations between the identified proteins and diseases, the dataset was subjected to DAVID Genetic Association Database (GAD) analysis.

The cBio Cancer Genomics Portal^2^ was used to determine the association of selected proteins with prognosis of PC using The Cancer Genome Atlas (TCGA) on pancreatic adenocarcinoma (access date, February 25, 2020). Our search was set to a total of 184 pancreatic adenocarcinoma samples (TCGA, PanCancer Atlas) and mRNA expression with a *z*-score threshold ±2.0. The role of selected candidates in cancer development was explored via the Cancer Hallmarks Analytics Tool, which allows organization and classification of cancer-related literature based on a text-mining analysis of 26 million PubMed abstracts (24).

### Statistical Analysis

Statistical analysis was conducted using the GraphPad Prism software version 8.0 (GraphPad Software Inc., La Jolla, CA, United States). Statistical significance was determined by one-way analysis of variance (ANOVA). *p* < 0.05 was set the level of statistical significance. Data are presented as mean, with error bars representing the standard deviation. *p-*value is displayed as ^∗^*p* < 0.05, ^∗∗^*p* < 0.01, ^∗∗∗^*p* < 0.001.

## Results

### Protein Map of SC Secretome and Pathway Analysis

**FIGURE 2:**
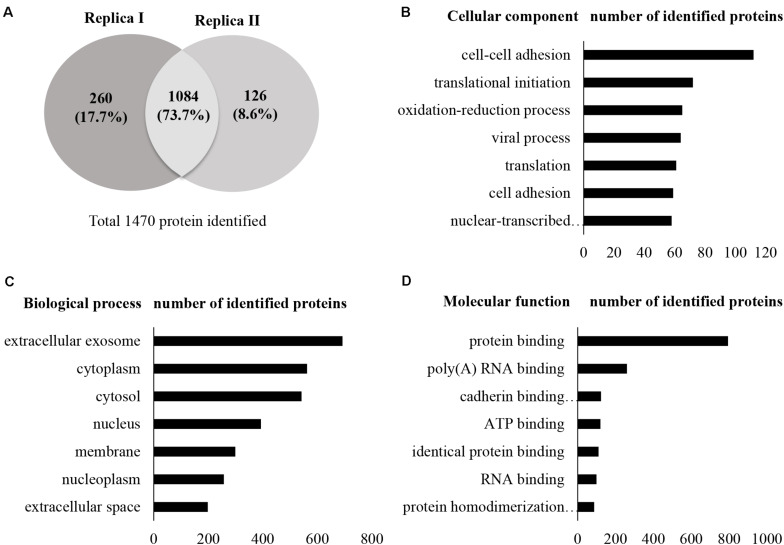
Secretome profile and functional clustering of human SCs. **(A)** Venn diagram summarizing the overlap in protein identification between two independent proteomic analyzes of SC-CM, which identified 1,344 (replica I) and 1,210 (replica II) proteins, respectively. Additional details are listed in Supplementary Tables S2–S4. **(B–D)** DAVID bioinformatics resource was used to analyze the secretome of SCs in order to identify enriched biological terms in the common secreted proteins extracted from the MS analysis. **(B)** GO enrichment analysis of cellular components. Proteins localized in extracellular exosomes were highly enriched. **(C)** GO enrichment analysis of biological processes. Highly enriched SC secreted proteins are involved in cell–cell adhesion. **(D)** GO enrichment analysis of molecular function. Highly enriched SC secreted proteins are involved in protein binding. Only the top seven cellular components, biological processes, and molecular functions have been shown here for vertical sizing. Additional details are listed in Supplementary Tables S5–S7. SC-CM, Schwann cell–conditioned media; GO, Gene Ontology; DAVID, Database for Annotation, Visualization, and Integrated Discovery.

**FIGURE 3:**
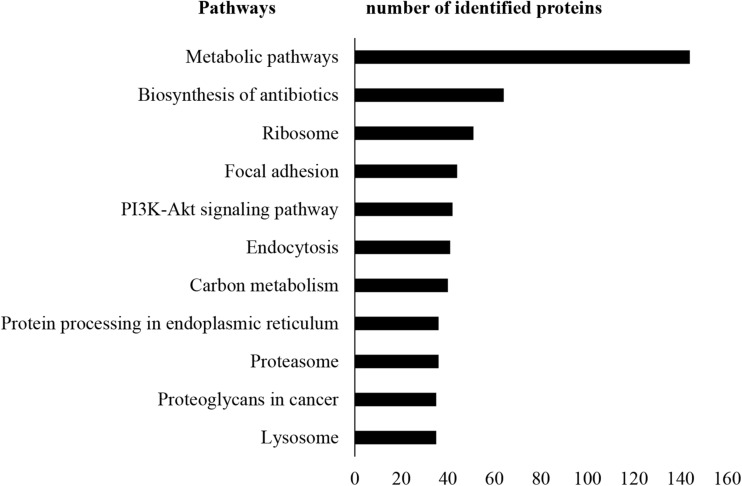
KEGG pathway analysis. KEGG pathway enrichment analysis was performed using DAVID. Metabolic pathways were found as most enriched. Only the top 10 enriched pathways are shown here for vertical sizing. Additional details are listed in Supplementary Table S8. KEGG, Kyoto Encyclopedia of Genes and Genomes; DAVID, Database for Annotation, Visualization, and Integrated Discovery.

A total of 13,796 unique peptides corresponding to 1,470 individual proteins were identified in two replicates, with a confidence corresponding to a false discovery rate <1% (Figure 2A and Supplementary Table S2–S4). Of the 1,470 proteins identified, 74% (1,084 proteins) were common across the two replicates. According to DAVID GO enrichment analysis of the cellular components, proteins localized in extracellular exosomes were highly enriched (692 proteins). Others were in cytoplasm (562 proteins), cytosol (542 proteins), nucleus (393 proteins), membrane (299 proteins), and nucleoplasm (257 proteins), as well as the extracellular space (198 proteins) (Figure 2B and Supplementary Table S5). To gain further insights into the enriched biological processes, common secreted proteins were subjected to GO enrichment analysis. The results showed that cell–cell adhesion was the most enriched (112 proteins) biological function. Other significant biological processes include translational initiation (72 proteins), oxidation–reduction process (65 proteins), and translation (61 proteins) (Figure 2C and Supplementary Table S6). GO enrichment analysis was also used to depict the molecular function of the identified proteins. Protein binding was the most enriched (794 proteins) molecular function. Other enriched molecular functions were mainly related to poly (A) RNA binding (260 proteins), cadherin binding (123 proteins), and ATP binding (120 proteins) (Figure 2D and Supplementary Table S7). KEGG pathway analysis was performed to map the important and representative pathways in human SCs using the DAVID resource, and the top 10 pathways based on enrichment were defined (Figure 3 and Supplementary Table S8). Metabolic pathways were among the most enriched (144 proteins). Proteins from our list were also found to be potentially involved in focal adhesion (44 proteins), PI3K-Akt signaling pathway (42 proteins), endocytosis (41 proteins), protein processing in endoplasmic reticulum (36 proteins), and proteoglycans in cancer (35 proteins). Exploration of potential associations of the identified proteins with diseases using GAD resources revealed that approximately 21% (226 proteins) of the SC-secreted proteins were associated with cancer followed by neurological disorders (204 proteins) and infectious diseases (177 proteins). The remaining portion of our identified proteins was also associated with renal disease (113 proteins), aging (93 proteins), reproduction (73 proteins), and vision (58 proteins) (Supplementary Figure S2 and Supplementary Table S9).

### Protein Validation by WB

**FIGURE 4:**
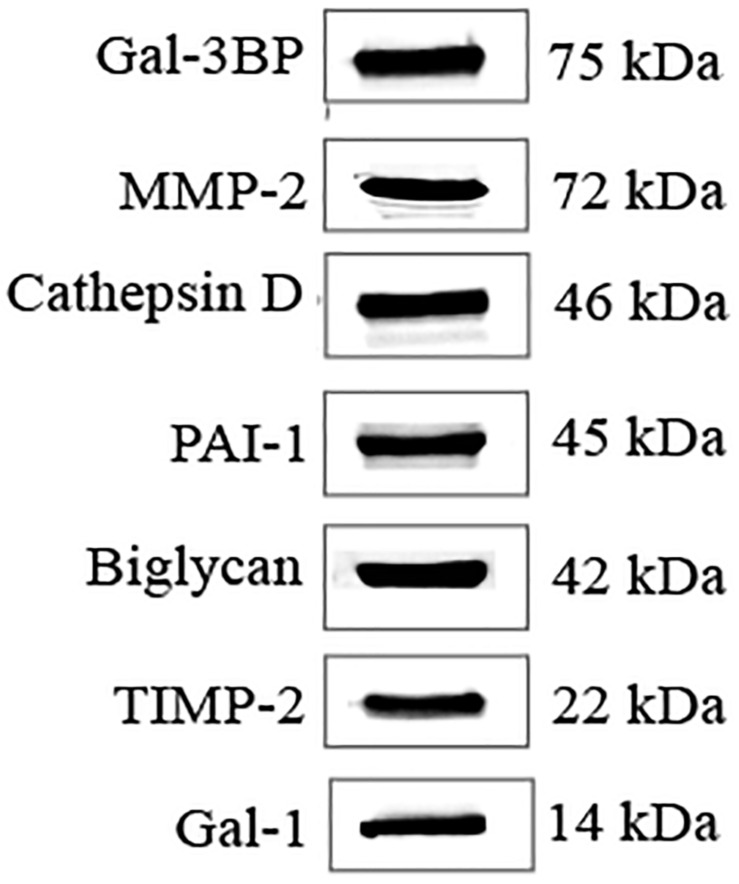
WB validation of candidate molecules. WB analysis confirmed the presence of secreted candidate molecules in SC-CM. WB, western blot; SC-CM, Schwann cell–conditioned media; Gal-3BP, galectin-3 binding protein; MMP-2, matrix metalloproteinase-2; PAI-1, plasminogen activator inhibitor-1; TIMP-2, tissue inhibitor of metalloproteinases-2; Gal-1, galectin-1.

From the list of secretory proteins identified by LC-MS/MS, galectin-3–binding protein (Gal-3BP), matrix metalloproteinase-2 (MMP-2), cathepsin D, plasminogen activator inhibitor-1 (PAI-1), biglycan, tissue inhibitor of metalloproteinases-2 (TIMP-2), and galectin-1 (Gal-1) were validated by WB (Figure 4). We initially picked those proteins because they were known to be involved in tumor progression. These proteins are reported in Table 2 with their identified peptide count, coverage, and observed function in PC progression. WB analyses of SC lysates for the identified proteins have also been provided in Supplementary Figure S3. Validated proteins with their Cancer Hallmarks Analytics Tool analysis results are presented in Supplementary Figure S4.

**TABLE 2:** SCs secreted proteins whose targeting with antibodies resulted in a decreased PC cell proliferation and invasion.

No.	Accession no.	Protein name	Gene name	Unique peptides	MW (kDa)	Coverage (%)	Role in PC progression
1	Q08380	Gal-3BP	*LGALS3BP*	23	65.3	42.90	Proliferation
2	P08253	MMP-2	*MMP2*	40	73.8	61.81	Proliferation and invasion
3	P07339	Cathepsin D	*CTSD*	21	44.5	56.55	Proliferation and invasion
4	P05121	PAI-1	*SERPINE1*	41	45	62.68	Proliferation and invasion
5	P21810	Biglycan	*BGN*	18	41.6	53.53	Proliferation
6	P16035	TIMP-2	*TIMP2*	14	24.4	49.54	Proliferation
7	P09382	Gal-1	*LGALS1*	14	14.7	74.81	Proliferation and invasion

*Commonly secreted proteins (between two replicates) identified using LC-MS/MS and validated by WB are presented here. Unique peptides: number of different peptides found by MS analyses; molecular weight: found by MS analyses; coverage: percentage of related protein found by MS analyses. LC-MS/MS, liquid chromatography–tandem mass spectrometry; Gal-3BP, galectin-3 binding protein; MMP-2, matrix metalloproteinase-2; PAI-1, plasminogen activator inhibitor-1; TIMP-2, tissue inhibitor of metalloproteinases-2; Gal-1, galectin-1.*

### Impact of Targeting SC Secreted Proteins on PC Cell Growth

**FIGURE 5:**
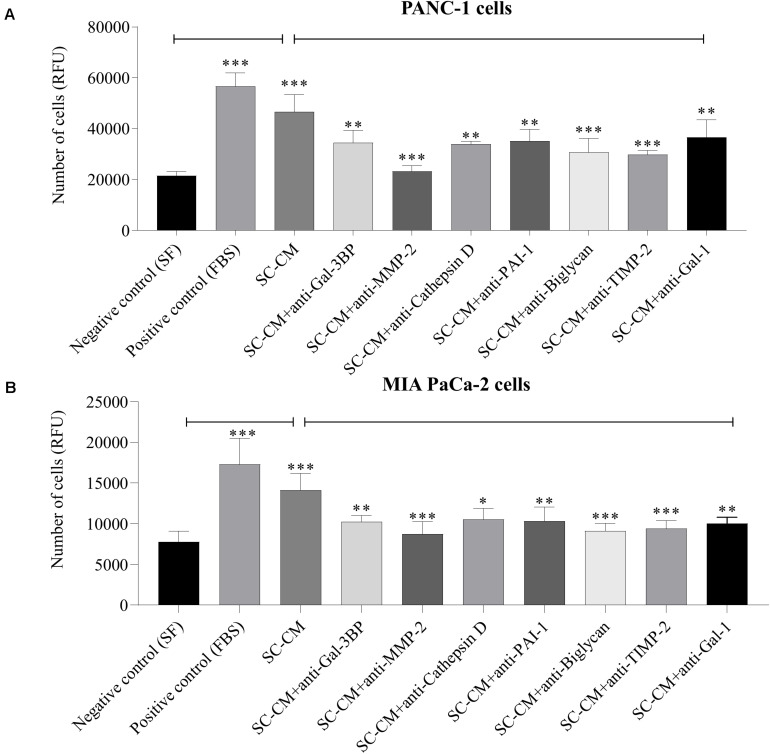
*In vitro* blockage of SC-secreted proteins results in decrease of PC cell proliferation. The effects of SC-CM on PC cells (PANC-1 and MIA PaCa-2) proliferation were studied *in vitro* using resazurin-based proliferation assay. For both treatment and control, ∼5,000 cells were initially plated onto 96-well plate dishes and allowed to proliferate for 72 h in the presence of SC-CM or control media. Serum-free media was used as negative control, and serum-supplemented media was used as positive control. Cell number was quantified as described in section “Materials and Methods.” A significantly greater number of PC cells were observed in presence of SC-CM compared to negative control (*p* < 0.05). To find out if the effect of increased proliferation of PC cells in presence of SC-CM was due to secreted proteins from SCs, proliferation assays were performed using blocking antibodies against the proteins of interest. Blocking antibodies were used at 6 μg/mL concentration. Significant decrease in cancer cell (both PANC-1 and MIA PaCa-2) proliferation was observed in presence of blocking antibodies against Gal-3BP, MMP-2, cathepsin D, PAI-1, biglycan, TIMP-2, and Gal-1 compared to SC-CM alone (*p* < 0.05). Representative data are shown from three independent experiments, performed in at least six replicates each. Statistical significance was confirmed by one-way ANOVA. The error bars represent the SD of the mean. *p-*values are displayed as ^∗^*p* < 0.05, ^∗∗^*p* < 0.01, and ^∗∗∗^*p* < 0.001. SC-CM, Schwann cell–conditioned media; PC, pancreatic cancer; Gal-3BP, galectin-3 binding protein; MMP-2, matrix metalloproteinase-2; PAI-1, plasminogen activator inhibitor-1; TIMP-2, tissue inhibitor of metalloproteinases-2; Gal-1, galectin-1; RFU, relative fluorescence unit.

Significant increase of PC cells (PANC-1 and MIA PaCa-2) proliferation was observed in the presence of SC-CM compared with negative control (SF media, noted as SF) (*p* < 0.05), and the increment was similar to that in the positive control (serum-supplemented media, noted as FBS) (Figure 5), demonstrating that SC-CM stimulates PC cell proliferation. To determine if the proliferative effect of SC-CM was due to specific SC-secreted proteins, proliferation assay was performed in the presence of blocking antibodies against the WB-validated proteins. Blocking antibodies against Gal-3BP, MMP-2, cathepsin D, PAI-1, biglycan, TIMP-2, or Gal-1 caused a significantly decreased (*p* < 0.05) proliferation in PC cells compared to control (SC-CM only, without blocking antibody) (Figures 5A,B). No significant inhibitory effect on PC cell proliferation was observed when control media (SF and serum-supplemented media) was treated with blocking antibodies (*p* > 0.05) (Supplementary Figure S5).

### Impact of Targeting SC Secreted Proteins on PC Cell Invasiveness

**FIGURE 6:**
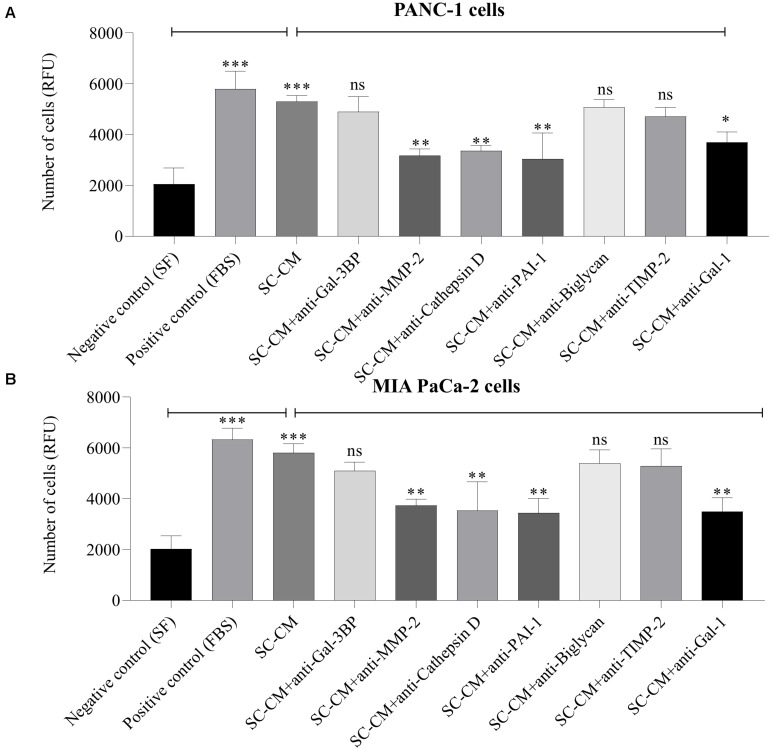
*In vitro* blockage of SC-secreted proteins results in decreased invasion of PC cells. The effects of SC-CM on PC cells (PANC-1 and MIA PaCa-2) invasiveness were studied *in vitro* using Transwell invasion assay. For both treatment and control, cells (∼60,000) were plated to the insert of a Transwell plate coated with collagen and allowed to invade for 24 h through a collagen-coated matrix toward SC-CM or control media as applicable. Serum-free media was used as negative control and serum-supplemented media was used as positive control. The non-invaded cells were removed from the top of the insert, and invaded cells were quantified using the QCM ECMatrix Cell Invasion Assay Kit described in section “Materials and Methods.” A significantly greater number of invaded PC cells in presence of SC-CM was observed compared to negative control (*p* < 0.05). To determine if the proteins of interest were involved in the stimulation of PC cells by SCs, the invasion assay was performed with blocking antibodies. Among the tested blocking antibodies, neutralization of MMP-2, cathepsin D, PAI-1, and Gal-1 decreased the invasiveness of both types of PC cell line compared to SC-CM alone (*p* < 0.05). Blocking antibodies were used at 6 μg/mL concentration. Blocking Gal-3BP, biglycan, and TIMP-2 in SC-CM had no significant inhibitory effect on PC invasiveness (*p* > 0.05). Representative data are shown from three independent experiments, performed in at least three replicates each. Statistical significance was obtained by one-way ANOVA. The error bars represent the SD of the mean. *p-*values are displayed as ^∗^*p* < 0.05, ^∗∗^*p* < 0.01, and ^∗∗∗^*p* < 0.001. SC-CM, Schwann cell–conditioned media; PC, pancreatic cancer; Gal-3BP, galectin-3 binding protein; MMP-2, matrix metalloproteinase-2; PAI-1, plasminogen activator inhibitor-1; TIMP-2, tissue inhibitor of metalloproteinases-2; Gal-1, galectin-1; RFU, relative fluorescence unit; ns, non-significant.

A significant increase in PC cell (both PANC-1 and MIA PaCa-2) invasion was observed in the presence of SC-CM compared with negative control (SF media, noted as SF) (*p* < 0.05), and the increment was similar to the positive control (serum-supplemented media, noted as FBS) (Figure 6). To specifically determine the SC-secreted proteins that induce increased invasion of PC cells, Transwell invasion assays were performed with blocking antibodies against the identified proteins. Among the blocking antibodies tested, neutralization of MMP-2, cathepsin D, PAI-1, and Gal-1 significantly (*p* < 0.05) decreased the invasion of PC cell lines compared to control (SC-CM only, without blocking antibody) (Figures 6A,B). In contrast, blocking Gal-3BP, biglycan, and TIMP-2 in SC-CM had no significant (*p* > 0.05) inhibitory effect on PC invasion. No significant decrease in PC cell invasiveness was observed when control media (both SF and serum-supplemented media) was treated with blocking antibodies (*p* > 0.05) (Supplementary Figure S6).

### Immunohistochemical Detection of Identified Proteins in PC

The seven SC-secreted proteins having an effect on PC cell growth and invasion were investigated by IHC. The results revealed moderate to high levels of expression of these proteins in PC (Supplementary Figures S7A–G). Briefly, strong cytoplasmic immunoreactivity was observed in case of Gal-3 BP (Supplementary Figure S7A). In case of MMP-2, most malignant cells and surrounding stroma showed weak to moderate immunoreactivity (Supplementary Figure S7B). Cathepsin D was more strongly found in the PC cells and in SCs (Supplementary Figure S7C). In case of PAI-1, all cancer tissues were negative, and few tumor stroma were weakly positive (Supplementary Figure S7D). In case of biglycan, strong tumor and stromal immunoreactivity was observed (Supplementary Figure S7E). In case of TIMP-2, most cancer tissues were negative, whereas few malignant cells displayed moderate cytoplasmic immunoreactivity (Supplementary Figure S7F). Gal-1 expression levels were mostly restricted to stroma of PC cells and in SCs (Supplementary Figure S7G). Positive labeling in IHC validates the presence of the identified proteins in the tumor microenvironment of PC. However, SCs are difficult to localize in the tumor microenvironment, and IHC cannot provide a clear demonstration that those proteins are released by SCs.

### Prognostic Value of Protein Candidates

**FIGURE 7:**
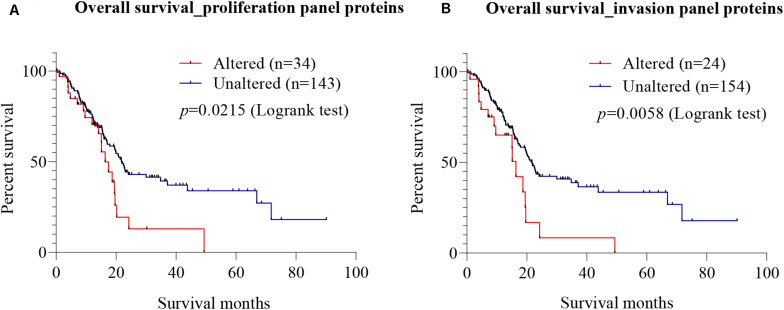
Prognostic value of mRNA expression corresponding to the identified candidate proteins. Using cBioportal, Kaplan–Meier estimates of survival for PC patients with alterations in different gene combination were performed using The Cancer Genome Atlas (TCGA) on pancreatic adenocarcinoma. **(A)** Survival analysis showed that patients with alterations in the seven-gene proliferation panel (Gal-3BP, MMP-2, cathepsin D, PAI-1, biglycan, TIMP-2, and Gal-1) had worse overall survival than those without alterations (log-rank test *p* = 0.0215). **(B)** In the cases of invasion panel proteins (MMP-2, cathepsin D, PAI-1, and Gal-1), worse prognosis was also observed for patients with alterations in the four genes than those without alterations (log-rank test *p* = 0.0058). PC, pancreatic cancer; Gal-3BP, galectin-3 binding protein; MMP-2, matrix metalloproteinase-2; PAI-1, plasminogen activator inhibitor-1; TIMP-2, tissue inhibitor of metalloproteinases-2; Gal-1, galectin-1.

To explore the prognostic value of candidate proteins for which targeting with antibodies inhibited proliferation and invasion of PC cells, a meta-analysis at the mRNA was carried out using TCGA on pancreatic adenocarcinoma, using the cBio Cancer Genomics Portal. Using Kaplan–Meier analysis of overall survival, based on the median mRNA expression levels, the prognostic values of the combined seven proteins involved in proliferation (Gal-3BP, MMP-2, cathepsin D, PAI-1, biglycan, TIMP-2, and Gal-1) (called proliferation protein panel) and four proteins involved in invasion (MMP-2, cathepsin D, PAI-1, and Gal-1) (called invasion protein panel) were investigated. The proliferation protein panel showed a significant association with poor prognosis (log-rank test *p*-value 0.0215), where 34 patients from a total of 177 showed alterations in this gene signature (Figure 7A). The invasion protein panel showed significant association with poor prognosis (log-rank test *p* = 0.0058), where 24 patients from a total of 178 showed alterations in this gene signature (Figure 7B). Overall, both protein panels may constitute a molecular signature for poor prognosis in PC. The data are useful in showing a prognostic relevance of the combination, but there is no demonstration that SCs are the major source for these proteins that may equally be produced by PC cells.

## Discussion

**FIGURE 8:**
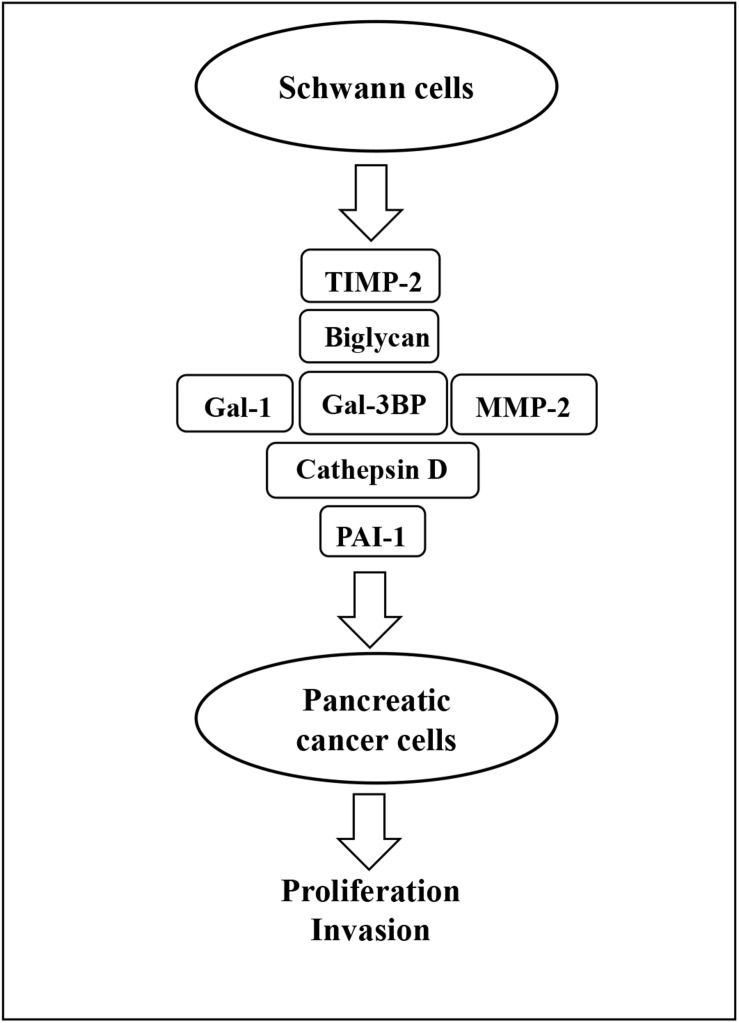
Schematic representation of SC-stimulation of PC cells. Gal-3BP, MMP-2, cathepsin D, PAI-1, biglycan, TIMP-2, and Gal-1 are the SC-secreted proteins that have been identified as potential promoters of PC cell proliferation and invasion in this study. SC, Schwann cell; PC, pancreatic cancer; Gal-3BP, galectin-3 binding protein; MMP-2, matrix metalloproteinase-2; PAI-1, plasminogen activator inhibitor-1; TIMP-2, tissue inhibitor of metalloproteinases-2; Gal-1, galectin-1.

The present study has used proteomic analysis to define the secretome of SCs and has identified several proteins that can be targeted *in vitro* to inhibit growth and invasion of PC cells. These findings are summarized in Figure 8. In addition, several of the identified proteins were shown to contribute to PC cell growth and invasion and may constitute future therapeutic targets.

The LC-MS/MS–based proteomic analysis that has been implemented in this study has enabled the identification of 1,084 SC-secreted proteins. Bioinformatics analysis was performed. In GO analysis, the “extracellular exosome” was found to be the most represented localization, and that is in accordance with the fact that the exosome compartment is a key part of the secretome. Cell–cell adhesion was the most enriched biological function, and this is in line with the supportive role of SC in nerves (25) and their role in promoting the nerve-cancer cell interaction (11). Strikingly, GO analysis also revealed that 73% of the identified proteins possessed molecular functions related to protein binding. Gal-3 BP or insulin-like growth factor BP (IGFBP), for instance, is well described for its binding activities that contribute to the regulation of cell growth (26, 27). The SC secretome was also enriched in proteins involved in molecular functions related to “catalytic activity,” which may play important roles in cancer progression. For instance, cathepsin D is a catalytic protein that stimulates cancer cell proliferation and tumor angiogenesis and can also provide protection against tumor apoptosis (28). Similarly, other significantly enriched molecular functions including receptor binding, fibroblast growth factor binding, and platelet-derived growth factor binding are all pertinent to tumorigenesis and metastasis. Collectively, these findings indicate that the protein signatures identified in the SC secretome match molecular networks and biological processes associated with tumor progression. In future studies, it would be of interest to investigate the proteome of SCs when stimulated by PC cells to further delineate the crosstalk between PC cells and SC in the tumor microenvironment.

Some of the identified proteins were validated by WB, and blocking antibodies were used to test the effect of their inhibition on PC cell proliferation and invasion *in vitro*. We observed that blocking antibodies against Gal-3BP, MMP-2, cathepsin D, PAI-1, biglycan, TIMP-2, and Gal-1 inhibited the proliferation of PC cells induced by SC-CM. Additionally, blocking MMP-2, cathepsin D, PAI-1, and Gal-1also reduced cancer cell invasion. The potential role of each of these proteins in PC cancer is discussed below.

Galectin-3 BP, also known as tumor-associated antigen 90K, is a large oligomeric heavily glycosylated and secreted protein (26). It is a binding partner of Gal-1 and Gal-3, which promote integrin-mediated cell adhesion, and significantly elevated expression of Gal-3 BP in the serum or tumor tissues is associated with poor prognosis in a variety of malignancies including breast cancer (29), lung cancer (30), and PC (31). Additionally, Gal-3 BP is involved in the promotion of integrin-mediated tumor cell adhesion to the ECM proteins in colon cancer (32), breast cancer (33), and the formation of metastasis in lung cancer (34). Our study reveals that targeting Gal-3 BP may prevent the stimulatory effect of SCs in PC cancer cells. In addition, Gal-1, a binding target of Gal-3 BP, has also been identified in our study.

Galectin-1 is a dimeric carbohydrate BP that facilitates the malignant cellular activities by cross-linking glycoproteins (35). It has been reported to play a role in cell invasion of several tumor types, including pancreatic (36), lung (37), and epithelial ovarian tumors (38). Knockdown of this protein can decrease the invasiveness of cancer cells in cervical cancer (39) and oral squamous cell carcinomas (40). Gal-1 has been reported to promote cancer cell invasion by enhancing the expression and enzymatic activities of MMP-2 and MMP-9 (40). It also appears to promote epithelial–mesenchymal transition in lung cancer cell lines (41), and our study points to the role of this protein in the SC-induced stimulation of PC cell growth and invasion.

Matrix metalloproteinase-2, a zinc-dependent endopeptidase, has been implicated in the malignant potential of tumor cells, because of its ability to degrade ECM proteins (42). MMP-2 is associated with the development of desmoplastic reaction in PC (43), and downregulation of MMP-2 reduces PC cell migration (44) and invasion (45). Our study reveals that MMP-2 is a potential mediator of the stimulatory role of SCs in PC cell proliferation and invasion. Interestingly, TIMP-2, tissue inhibitor of metalloproteinases (TIMPs) family, has also been identified in our study. TIMP-2 was described to decrease cell proliferation and migration *in vitro* via the inhibition of MMPs (46). However, it is associated with poor patient outcomes in cancers including gastric (47), renal (48), and oral squamous cell cancers (49). In lung cancer, TIMP-2 has been reported to inhibit tumor growth by promoting an antitumoral transcriptional profile both *in vitro* and *in vivo* (50). High expression of TIMP-2 has been shown to correlate with adverse prognosis in breast cancer (51). Our study indicates that MMP-2 and TIMP-2 are both released by SC and that they can contribute to the stimulation of PC cell growth and invasion. Similarly, PAI-1, another protease inhibitor also known as serpin (52), has also been identified in our study. It has been reported to inhibit proliferation of hepatocellular (53) and prostate cancer cell growth (54). In ovarian cancer, PAI-1 facilitates cell growth and inhibits apoptosis (55). Overexpression of PAI-1 inhibited cell migration and invasion in PC (56). Our study shows that PAI-1 is secreted by SC and can stimulate PC cell proliferation and invasion.

Cathepsin D is a secreted aspartic protease, which when highly expressed is associated with unfavorable clinical outcomes in patients with PC (57). It has been shown that cathepsin D expression can accelerate the metastatic spread of PC by upregulation of S100P (58). Combination of cathepsin D with CA-19-9 and MMP-7 has been reported to be an important panel of markers for screening PC (59). High cathepsin D expression in PC has been shown to decrease the effectiveness of adjuvant gemcitabine (60). The present study reveals the potential role of cathepsin D as a promoter of PC cell proliferation and invasion induced by SC.

Biglycan is a leucine-rich proteoglycan whose overexpression is related to enhanced angiogenesis and tumor invasion (61). Correlations of biglycan expression with aggressive clinicopathological features and poor survival in human cancers such as pancreatic adenocarcinoma (62), colorectal cancer (61), and gastric cancer (63) have been reported. High biglycan expression has been shown to promote invasiveness of melanoma cells (64). It has been reported to promote tumor invasion, migration, and metastasis of gastric cancer cells both *in vitro* and *in vivo* through activating the FAK signaling pathway (63). Our results suggest that biglycan is secreted by SC and can stimulate PC cell proliferation.

Our proteomic analysis also identified several other proteins of interest that, although they were not tested in functional analysis, may still be important in the stimulation PC cells by SCs. This is, for instance, the case of IGFBPs (IGFBP-2, IGFBP-4, IGFBP-5, IGFBP-6, IGFBP-7) and transforming growth factors (TGF-β1, TGF-β2, TGF-BI). Further studies are warranted to determine the possible involvement of these proteins in cancer PC progression and their potential value as therapeutic targets.

## Conclusion

In summary, this proteomic and functional analysis identified a number of SC-secreted proteins that seem to be involved in the proliferation and invasiveness of PC cancer cells induced by SC. Further preclinical studies *in vivo* are warranted to determine whether these proteins may become new targets for therapeutic intervention in PC.

## Data Availability Statement

The original contributions presented in the study are publicly available. This data can be found here: the Mass Spectrometry Interactive Virtual Environment (MassIVE) database with the dataset identifier MSV000084303 (https://massive.ucsd.edu/ProteoSAFe).

## Author Contributions

HH and AF designed the study and wrote the manuscript. AF and XL carried out the experiments. AF analyzed the data. XL, NG, SF, MJ, FG, CJ, DH, PT, and PJ edited the manuscript. All authors contributed to manuscript revision, read, and approved the submitted version.

## Conflict of Interest

The authors declare that the research was conducted in the absence of any commercial or financial relationships that could be construed as a potential conflict of interest.

## Footnotes

1 https://david.ncifcrf.gov/

2 http://cbioportal.org

## Abbreviations

cat. no.: catalog number
CM: conditioned media
DAVID: Database for Annotation Visualization and Integrated Discovery
ECM: extracellular matrix
GAD: Genetic Association Database
Gal-1: galectin-1
Gal-3BP: galectin-3 binding protein
GO: Gene Ontology
IGFBP: insulin-like growth factor-binding proteins
IHC: Immunohistochemistry
KEGG: Kyoto Encyclopedia of Genes and Genomes
LC-MS/MS: liquid chromatography–tandem mass spectrometry
MMP-2: matrix metalloproteinase-2
PAI-1: plasminogen activator inhibitor-1
PC: pancreatic cancer
RFU: relative fluorescence unit
SC: Schwann cell
SCM: Schwann cell medium
SD: standard deviation
SF: serum-free
TCGA: The Cancer Genome Atlas
TIMP-2: tissue inhibitor of metalloproteinases-2
TMA: tissue micro array
WB: western blot.
