# Supplementary material for: Schwann Cell Stimulation of Pancreatic Cancer Cells: A Proteomic Analysis
Source: Front Oncol. 2020 Aug 25;10:1601. doi: 10.3389/fonc.2020.01601 (PMC7477957; doi:10.3389/fonc.2020.01601)
Supplement: Supplementary file 1 [file Data_Sheet_1.PDF]

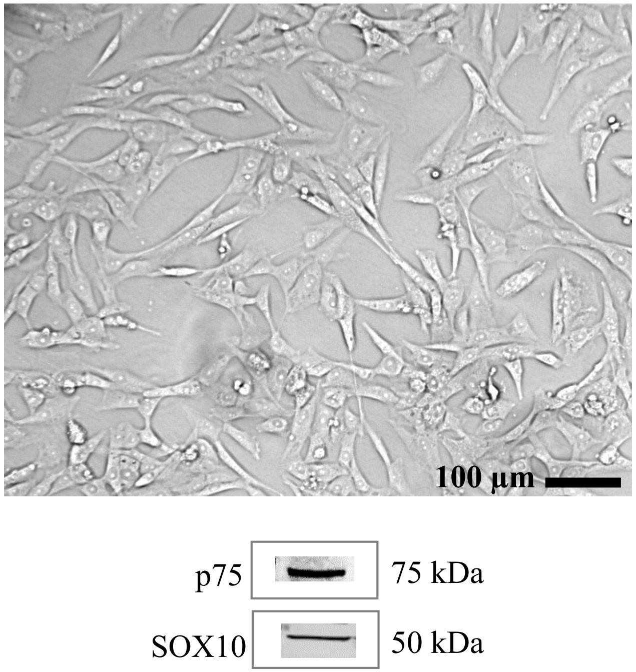

SUPPLEMENTARY FIGURE S1 | Representative image of human SCs. Cells show distinctive SC phenotype such as spindle-shaped morphology. Growing SCs were characterized by immunoblotting using antibodies against SC marker proteins p75 and SOX10 . Scale bar = 100µm. Abbreviation: SC, Schwann cell.

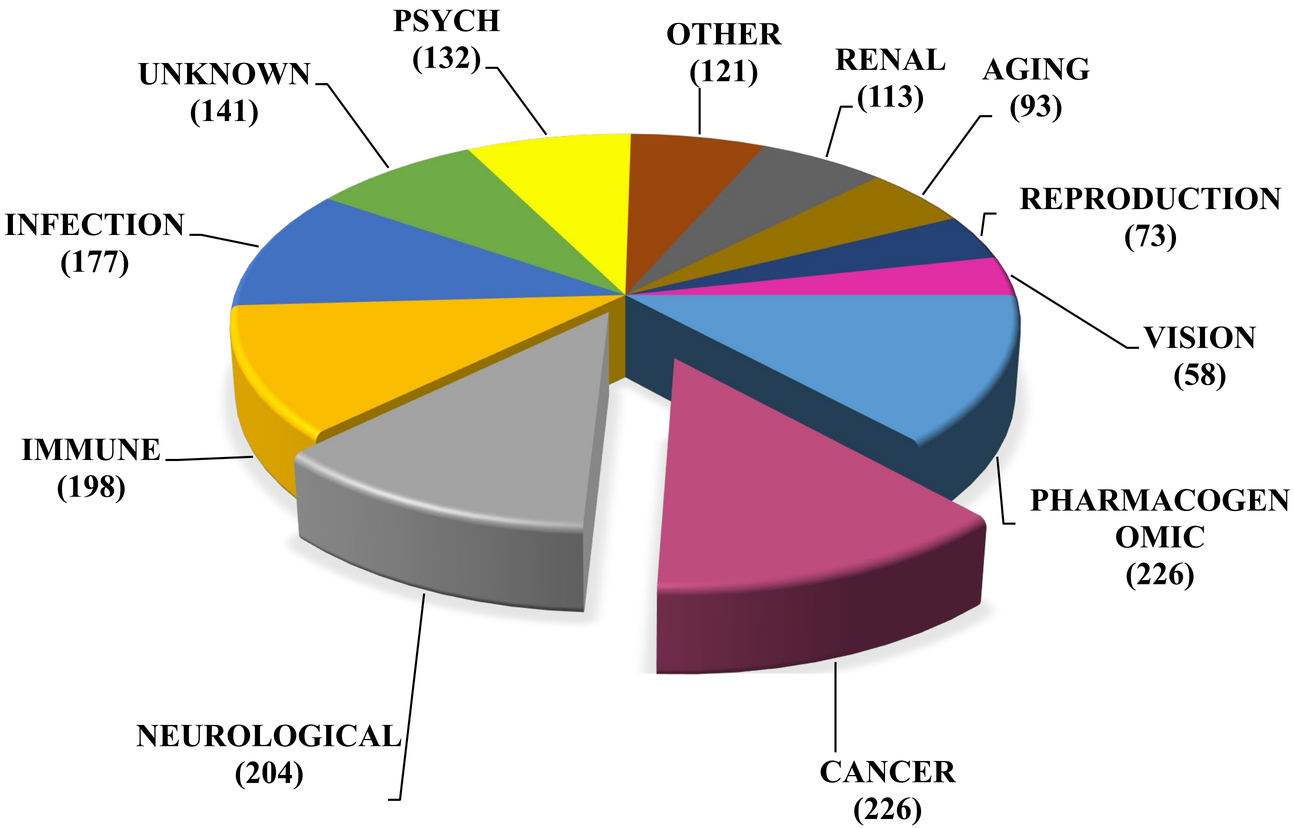

SUPPLEMENTARY FIGURE S2 | Distribution of the identified proteins based on disease classes. GAD analysis was performed using DAVID to investigate the potential association of the identified proteins with diseases. Number of proteins involved in different disease classes are displayed within brackets (multiple protein entries possible). Additional details are listed in Supplementary Table S9. Abbreviations: GAD, Genetic Association Database; DAVID, Database for Annotation, Visualization and Integrated Discovery.

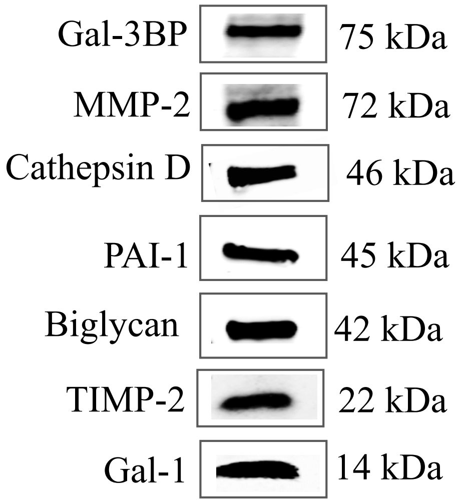

SUPPLEMENTARY FIGURE S3 | WB confirmation of candidate molecules in SC-lysate. WB analysis confirmed the presence of candidate molecules in SC-lysate. Abbreviations: WB, western blot; SC, Schwann cell; Gal-3BP, galectin-3 binding protein; MMP-2, matrix metalloproteinase-2; PAI-1, plasminogen activator inhibitor-1; TIMP-2, tissue inhibitor of metalloproteinases-2; Gal-1, galectin-1.

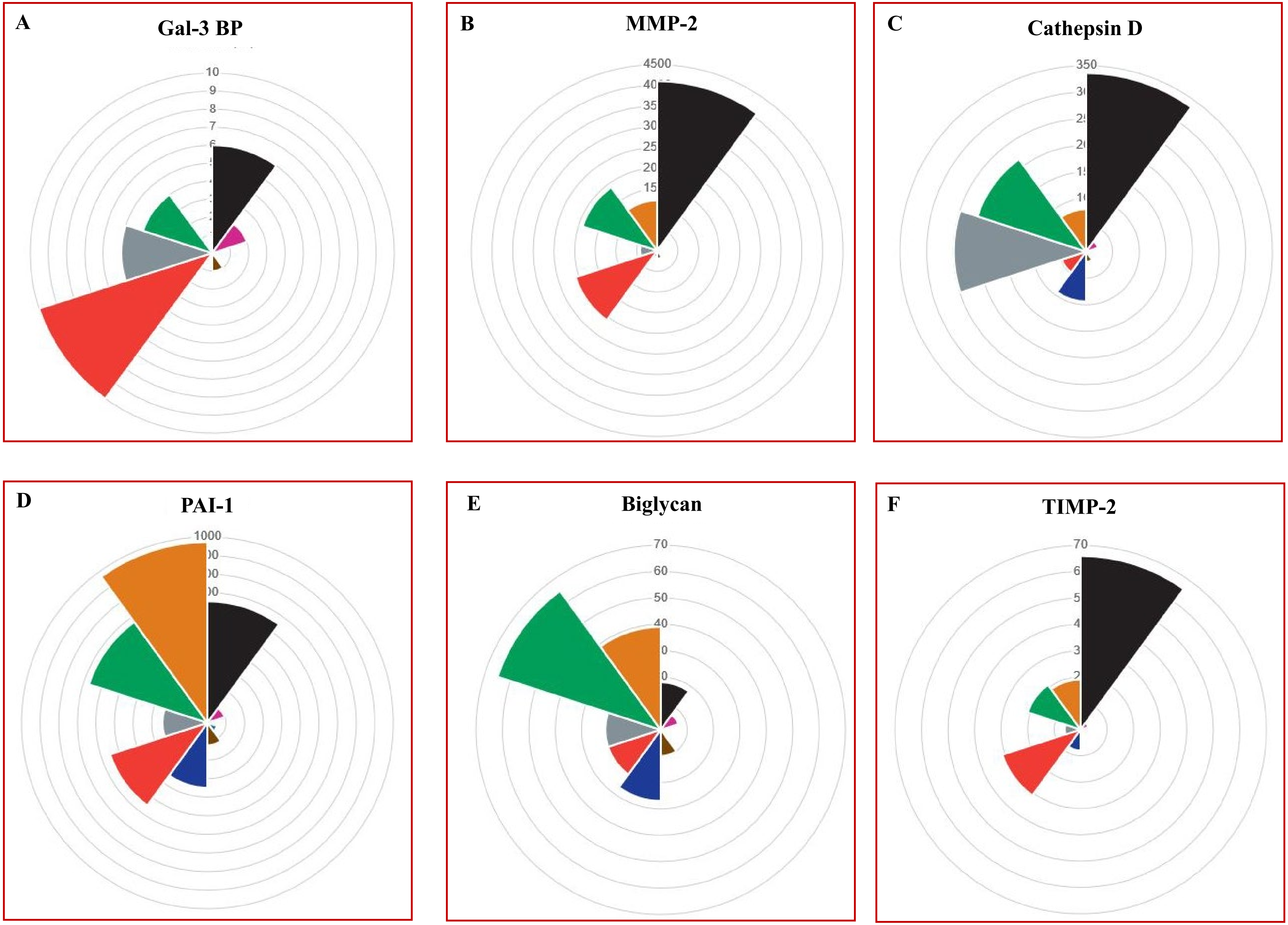
A
Gal-3 BP
10
9
8
7
B
MMP-2
4500
C
Cathepsin D
350
D
PAI-1
1000
E
Biglycan
70
60
50
40
F
TIMP-2
70

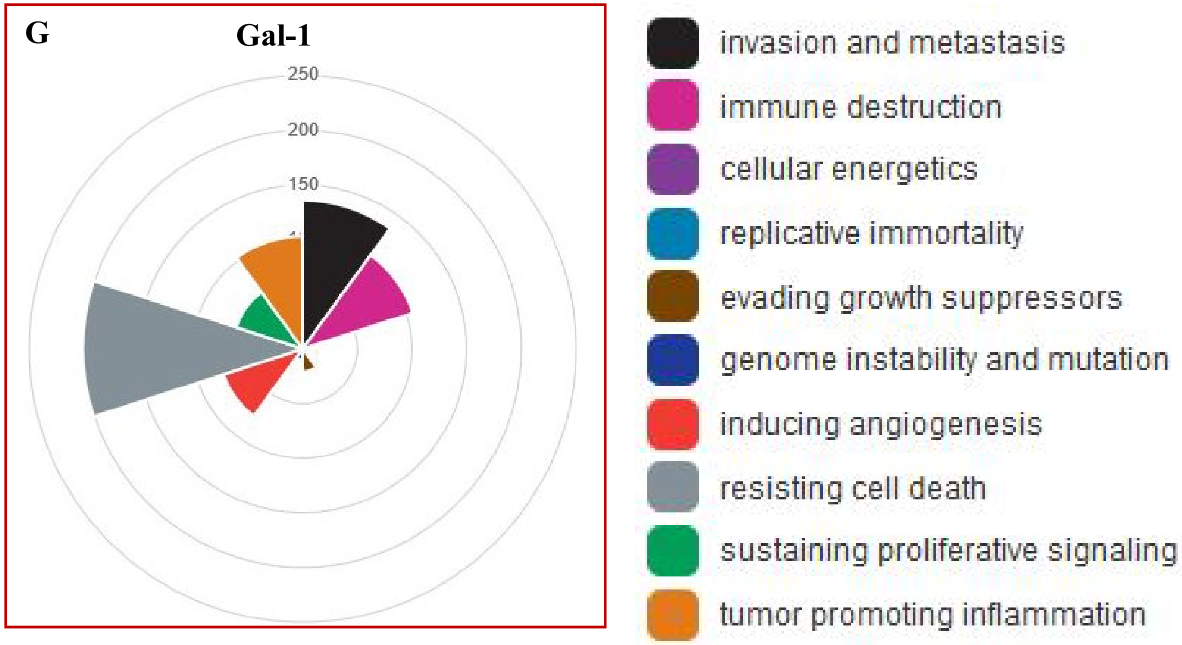

SUPPLEMENTARY FIGURE S4 | Role of candidate molecules in cancer progression. Investigations of the candidate molecules in cancer research using the Cancer Hallmarks Analytics Tool (CHAT) mainly focus on their crucial role in invasion and metastasis, sustaining proliferative signalling, inducing angiogenesis, tumour promoting inflammation and immune destruction in cancer development. CHAT can be accessed at: http://chat.lionproject.net. Abbreviations: Gal-3BP, galectin-3 binding protein; MMP-2, matrix metalloproteinase-2; PAI-1, plasminogen activator inhibitor-1; TIMP-2, tissue inhibitor of metalloproteinases-2; Gal-1, galectin-1.

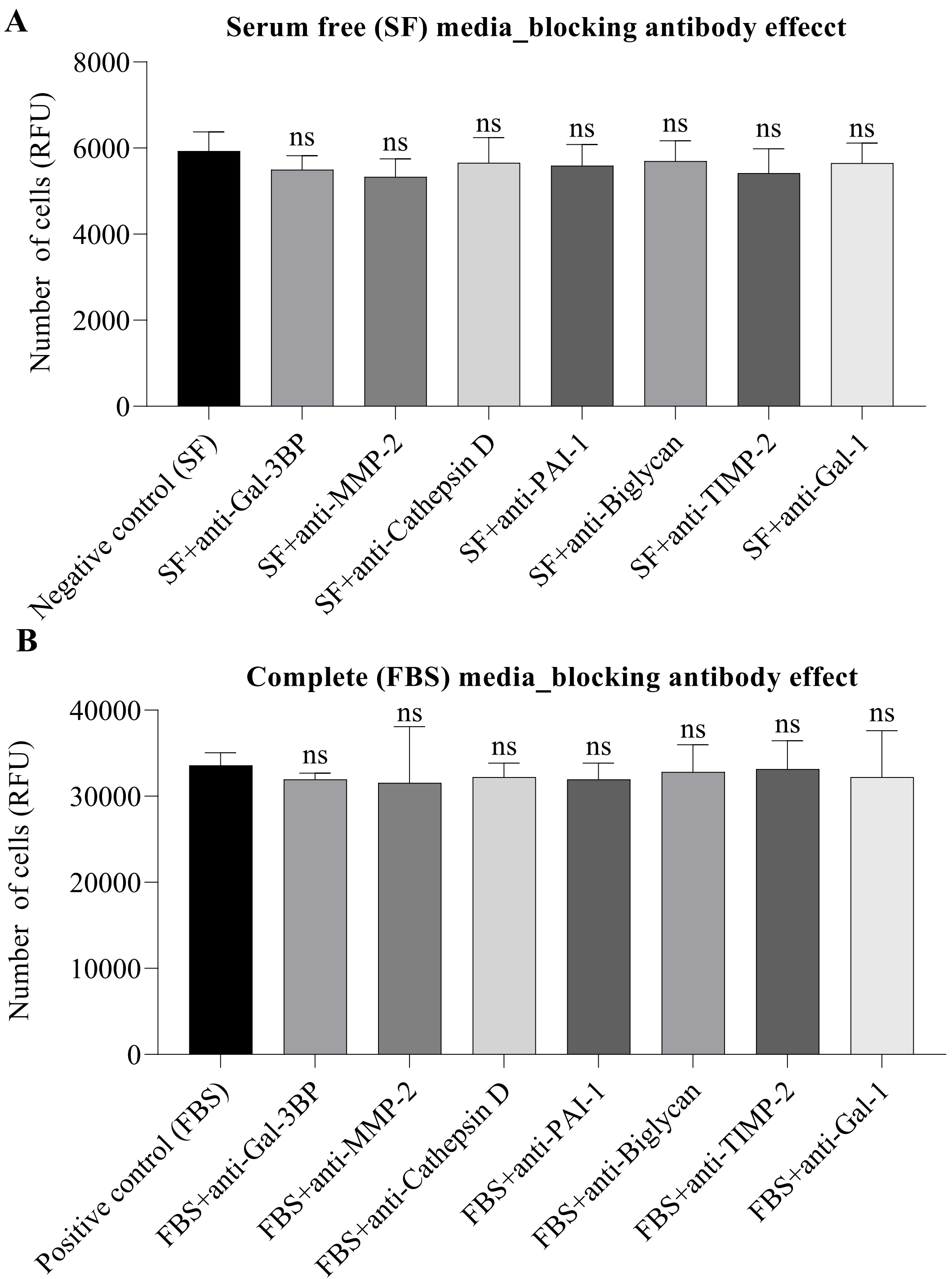

SUPPLEMENTARY FIGURE S5 | Blocking antibodies did not change the proliferative effect induced by SF or complete media on PC cells. To find out if the effect of increased proliferation of PC cells in presence of SC-CM was due to secreted proteins from SCs, or blocking antibodies themselves have any effect in absence of SC-CM, proliferation assays were performed using blocking antibodies against the proteins of interest. No significant decrease in PC cell (MIA PaCa-2) proliferation was observed in presence of blocking antibodies compared to either negative control (SF media only, **Figure A**) or positive control (serum supplemented media only, denoted FBS, **Figure B)** (p>0.05). Blocking antibodies were used at 6 µg/ml concentration. Statistical significance was confirmed by one-way ANOVA. The error bars represent the SD of the mean. Abbreviations: SF, serum free; FBS, fetal bovine serum; SC-CM, Schwann cell-conditioned media; PC, pancreatic cancer; Gal-3BP, galectin-3 binding protein; MMP-2, matrix metalloproteinase-2; PAI-1, plasminogen activator inhibitor-1; TIMP-2, tissue inhibitor of metalloproteinases-2; Gal-1, galectin-1; *RFU, relative fluorescence* unit; ns, non-significant.

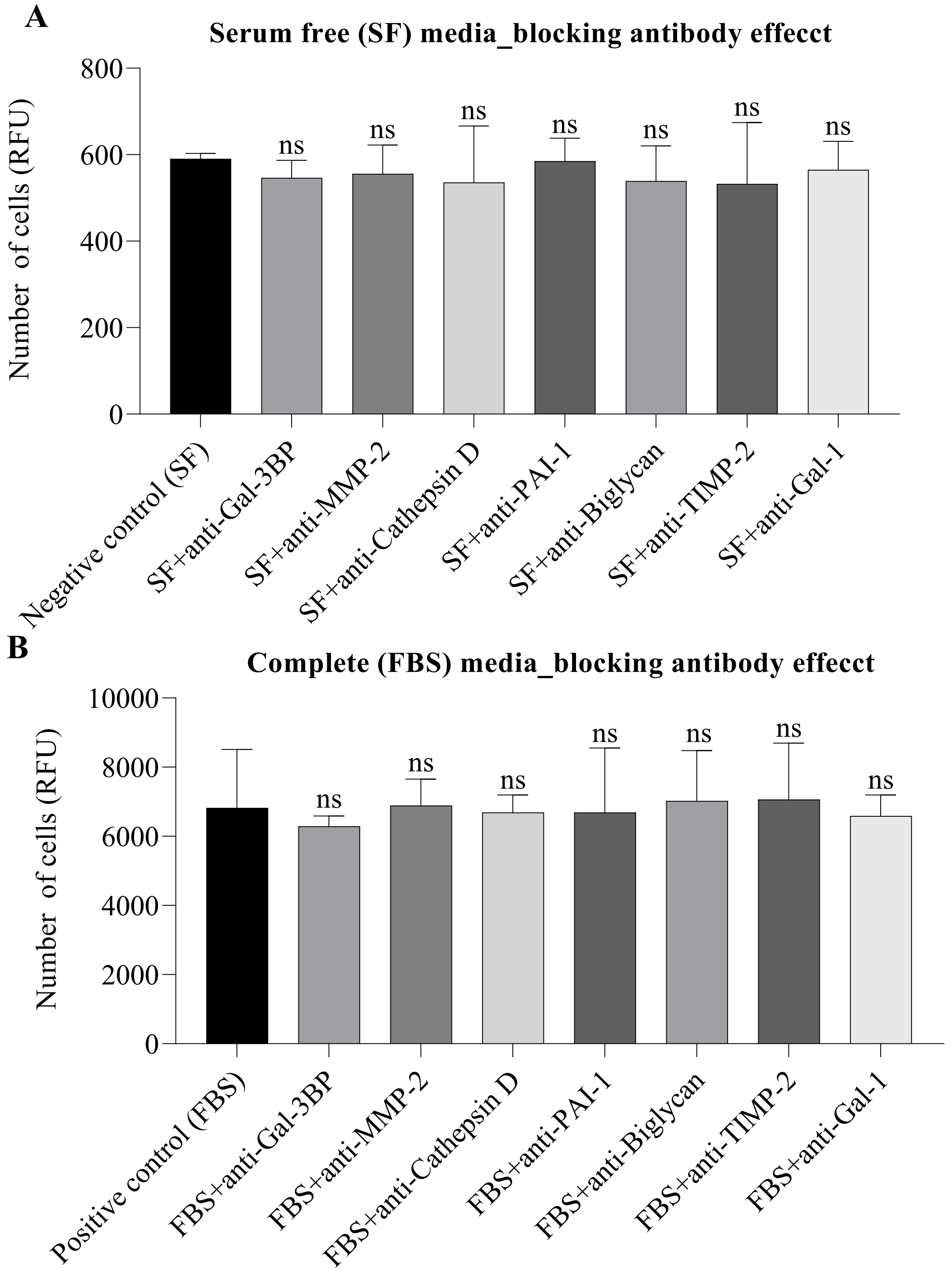

SUPPLEMENTARY FIGURE S6 | Blocking antibodies did not change the invasiveness induced by SF or complete media on PC cells. To find out if the effect of increased invasiveness of PC cells in presence of SC-CM was due to secreted proteins from SCs, or blocking antibodies themselves have any effect in absence of SC-CM, trans-well invasion assays were performed using blocking antibodies against the proteins of interest. No significant decrease in PC cell (MIA PaCa-2) invasiveness was observed in presence of blocking antibodies compared to either negative control (SF media only, **Figure A**) or positive control (serum supplemented media only, denoted FBS, **Figure B**) ($p>0.05$). Blocking antibodies were used at 6 μg/ml concentration. Statistical significance was confirmed by one-way ANOVA. The error bars represent the SD of the mean. Abbreviations: SF, serum free; FBS, fetal bovine serum; SC-CM, Schwann cell-conditioned media; PC, pancreatic cancer; Gal-3BP, galectin-3 binding protein; MMP-2, matrix metalloproteinase-2; PAI-1, plasminogen activator inhibitor-1; TIMP-2, tissue inhibitor of metalloproteinases-2; Gal-1, galectin-1; *RFU, relative fluorescence* unit; ns, non-significant.

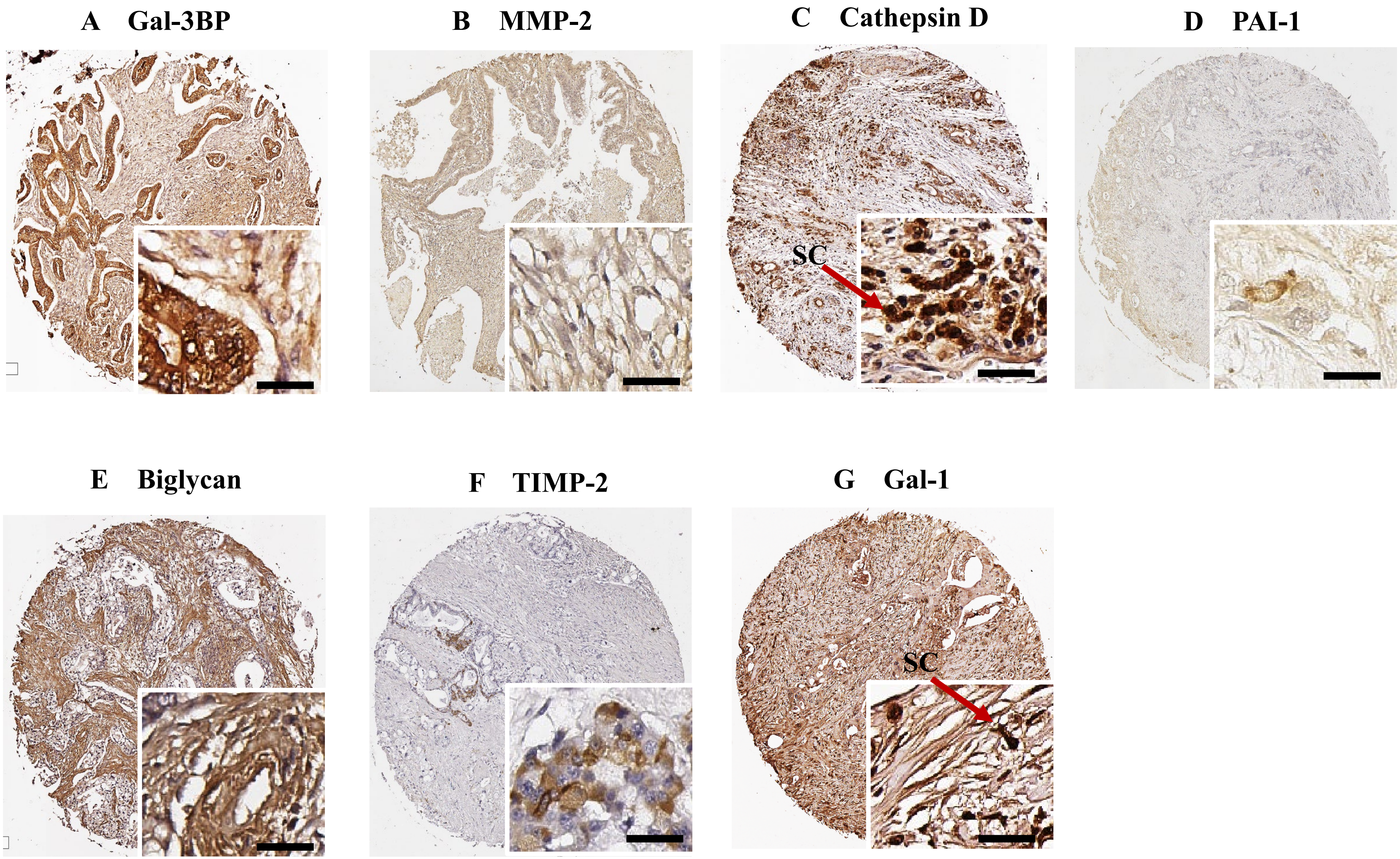

SUPPLEMENTARY FIGURE S7 | Expression of candidate proteins in clinical samples of PC. Immunohistochemical detection of seven candidate proteins was performed on a cohort of pancreatic cancer TMA revealing moderate to high levels of expression of the proteins. Representative images for each proteins are shown. SCs have been indicated by red arrow head. Scale bar: 200 μm. Abbreviations: PC, pancreatic cancer; SC, Schwann cell; TMA, tissue micro array; Gal-3BP, galectin-3 binding protein; MMP-2, matrix metalloproteinase-2; PAI-1, plasminogen activator inhibitor-1; TIMP-2, tissue inhibitor of metalloproteinases-2; Gal-1, galectin-1.
