# Supplementary material for: Schwann Cell Stimulation of Pancreatic Cancer Cells: A Proteomic Analysis
Source: Front Oncol. 2020 Aug 25;10:1601. doi: 10.3389/fonc.2020.01601 (PMC7477957; doi:10.3389/fonc.2020.01601)
Supplement: Supplementary file 2 [file Data_Sheet_2.PDF]

Supplementary material: Western blot full images

Gal-3 BP

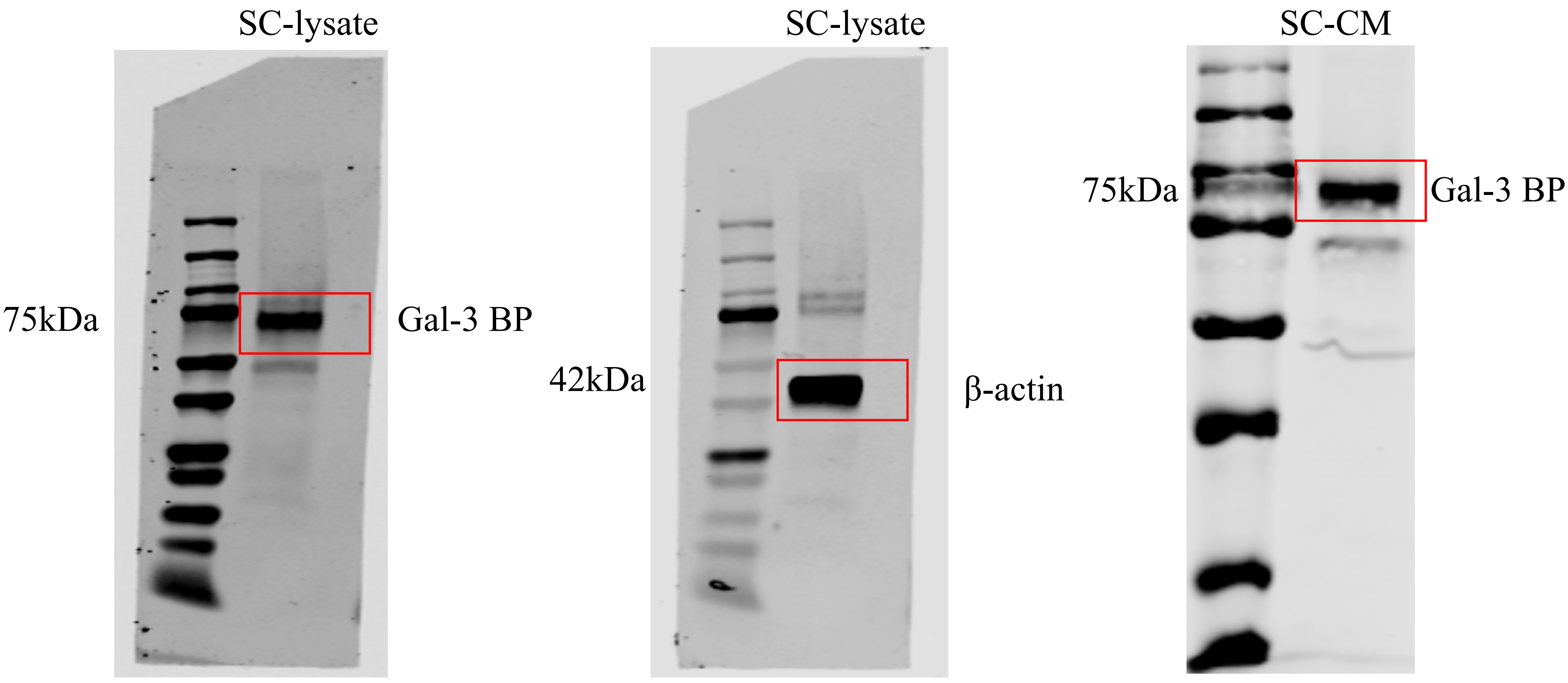

Gal-3BP, galectin-3 binding protein
SC-CM, Schwann cell-conditioned media

MMP-2

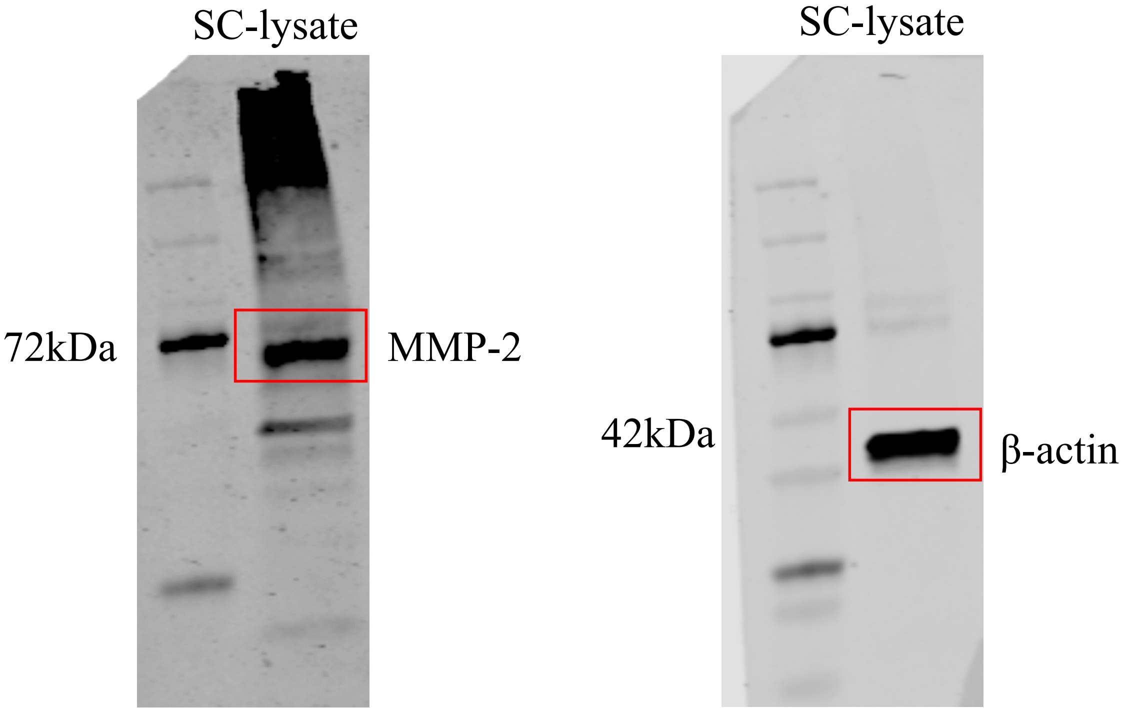

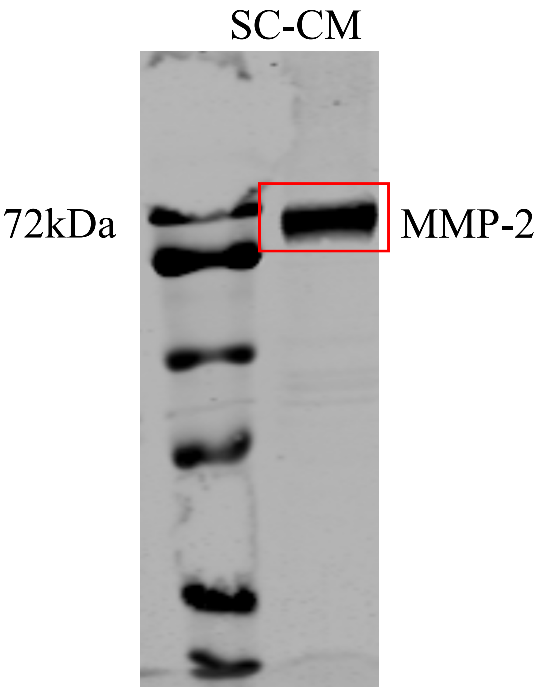

MMP-2, matrix metalloproteinase-2
SC-CM, Schwann cell-conditioned media

Cathepsin D

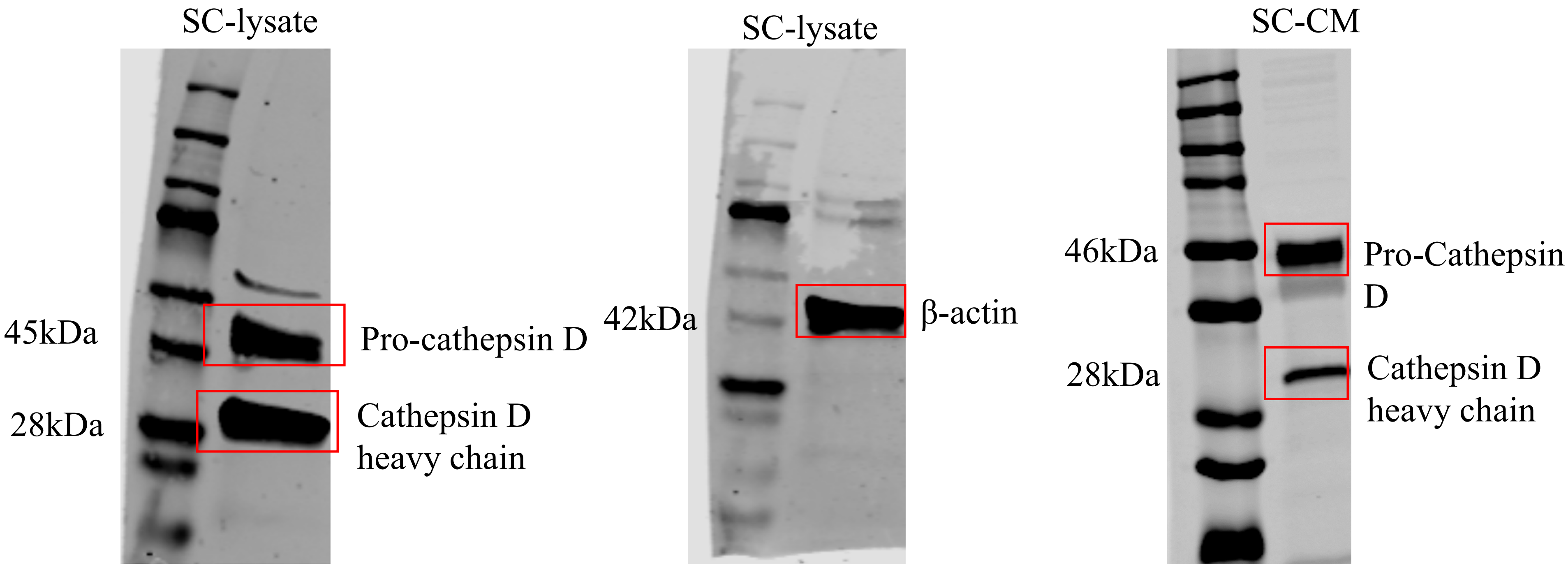

SC-CM, Schwann cell-conditioned media

PAI-1

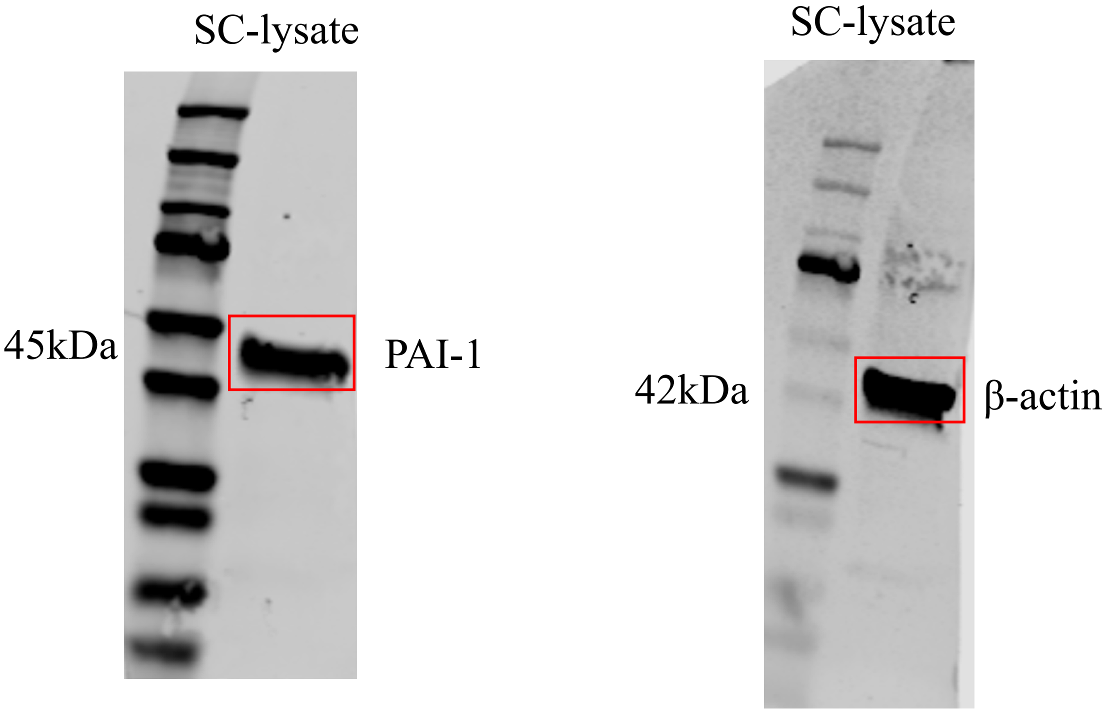

PAI-1, plasminogen activator inhibitor-1
SC-CM, Schwann cell-conditioned media

Biglycan

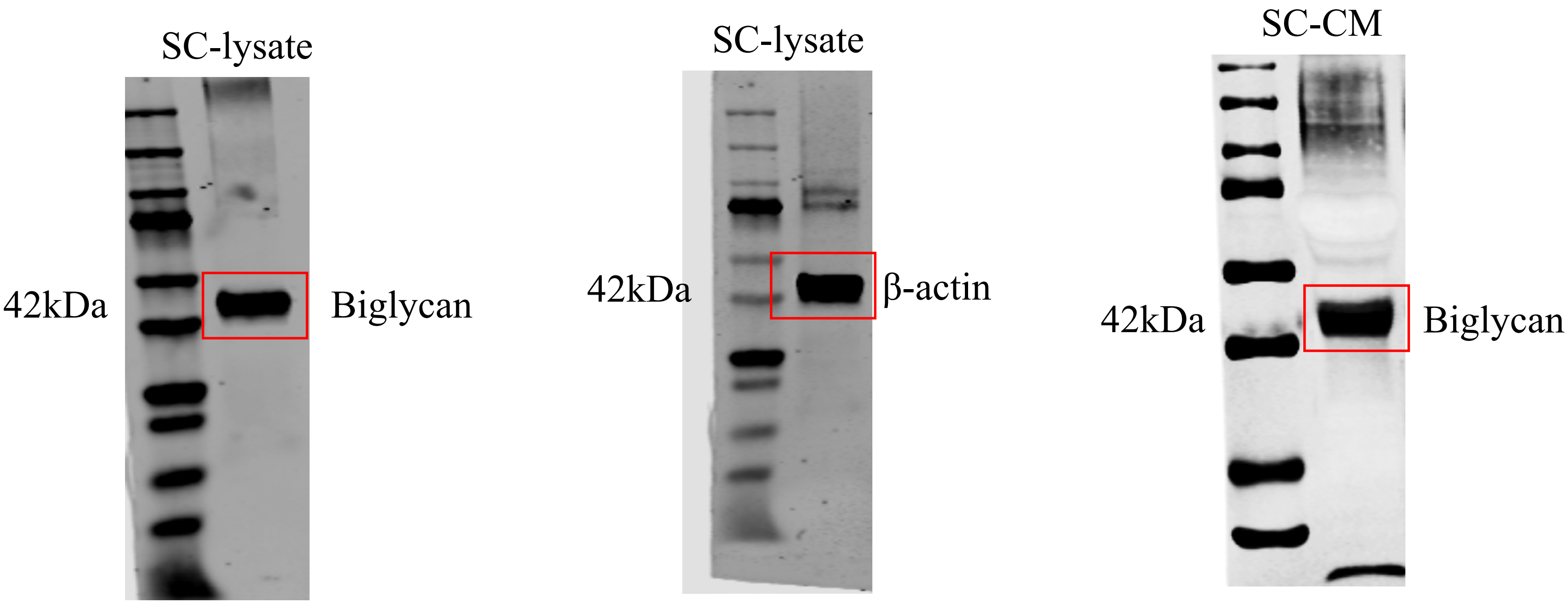

SC-CM, Schwann cell-conditioned media

TIMP-2

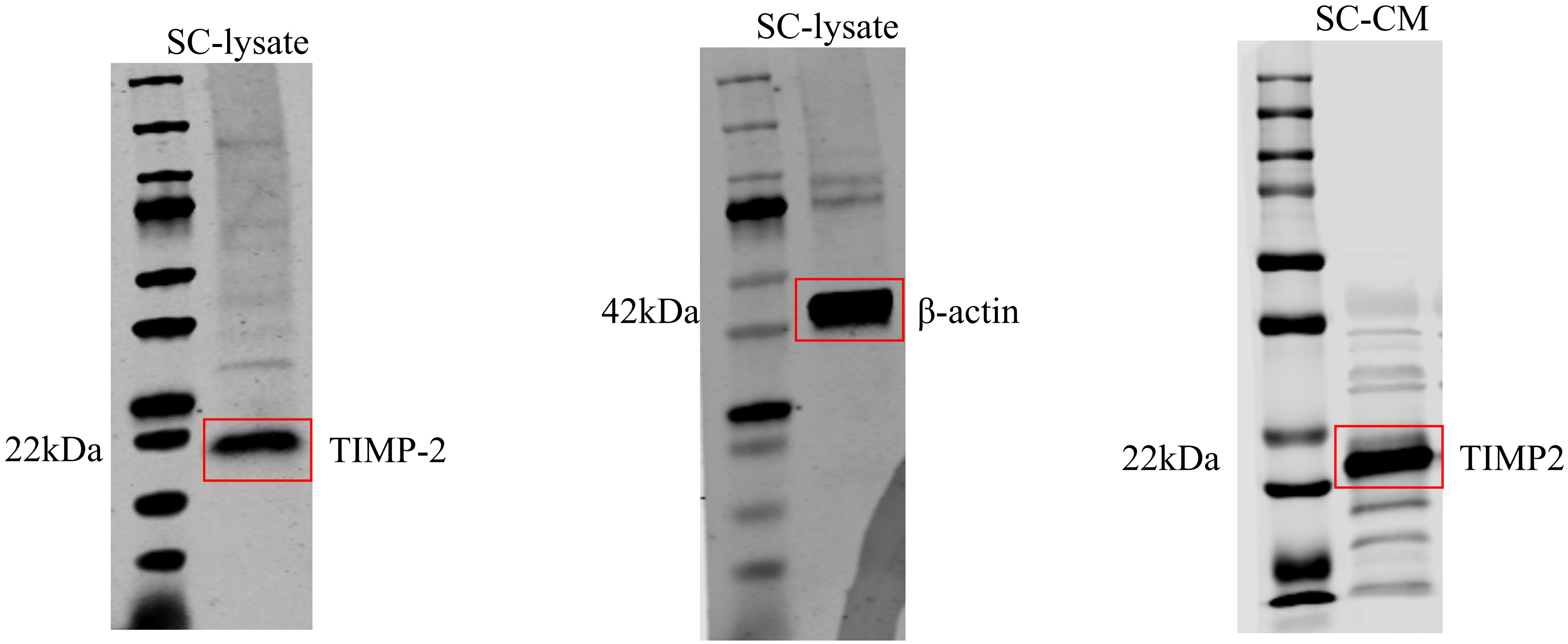

TIMP-2, tissue inhibitor of metalloproteinases-2
SC-CM, Schwann cell-conditioned media

Galectin-1

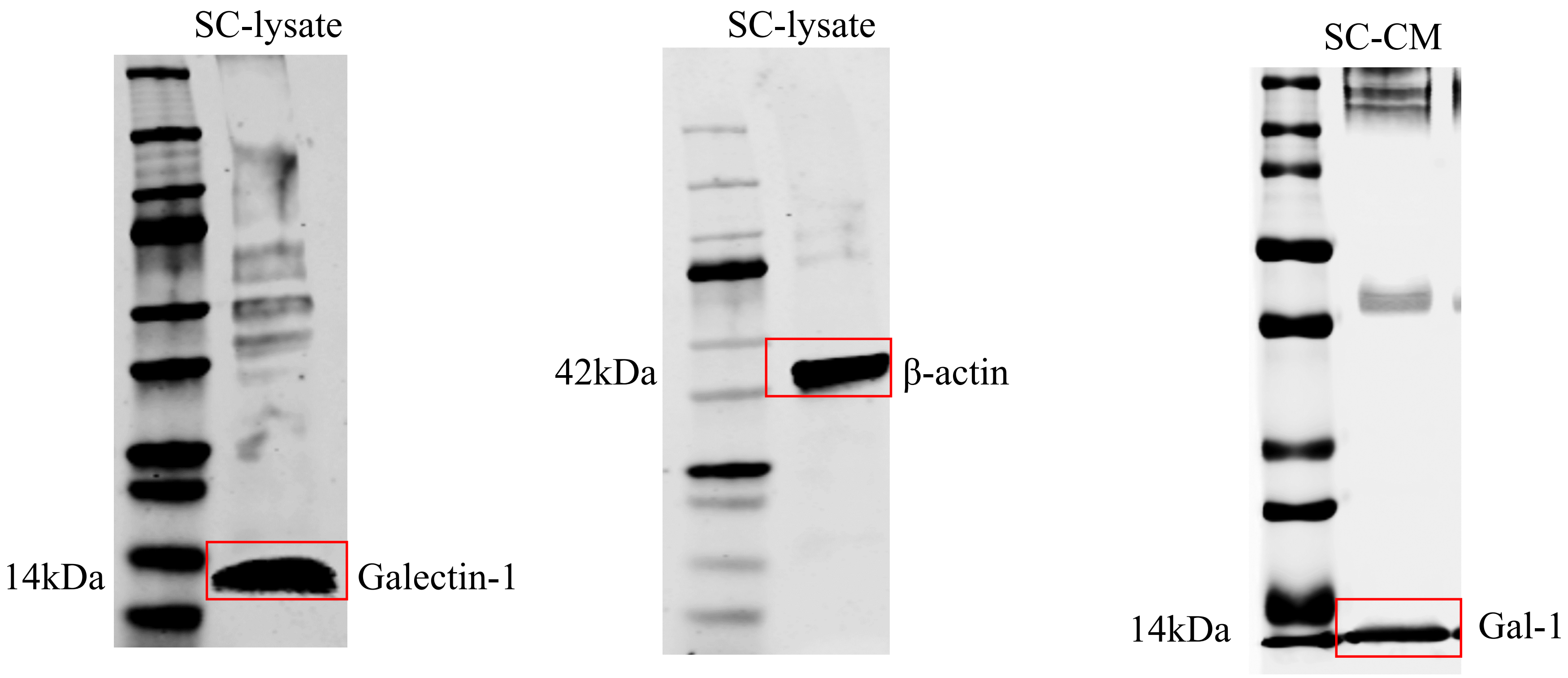

Gal-1, galectin-1
SC-CM, Schwann cell-conditioned media

Western blot full images: Schwann cell marker proteins

p75

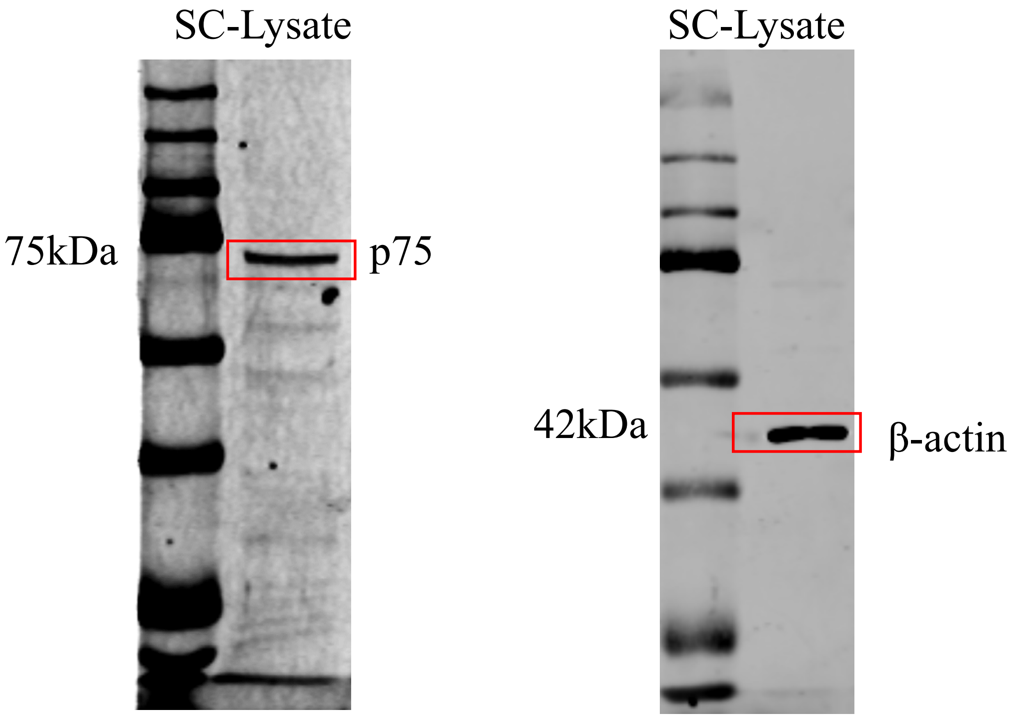

SC-Schwann cell

SOX10

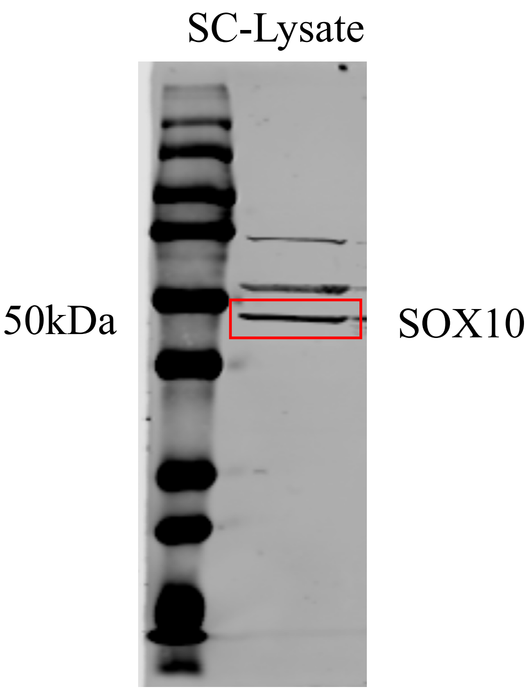

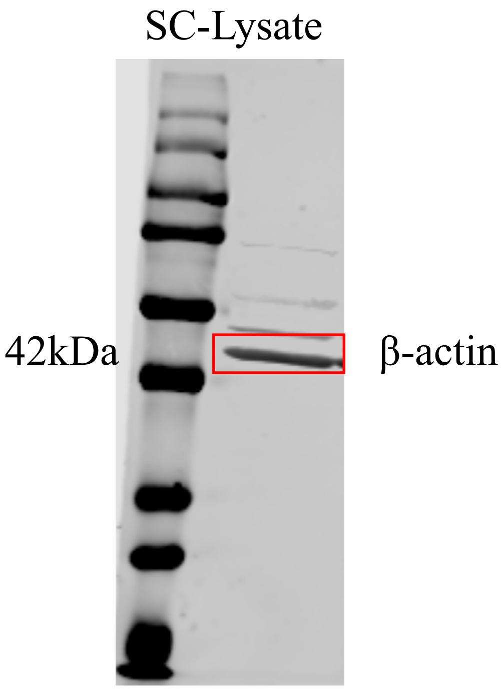

SC-Schwann cell
